# Changes in IPCC Scenario Assessment Emulators Between SR1.5 and AR6 Unraveled

**DOI:** 10.1029/2022GL099788

**Published:** 2022-10-18

**Authors:** Z. Nicholls, M. Meinshausen, J. Lewis, C. J. Smith, P. M. Forster, J. S. Fuglestvedt, J. Rogelj, J. S. Kikstra, K. Riahi, E. Byers

**Affiliations:** ^1^ International Institute for Applied System Analysis IIASA Laxenburg Austria; ^2^ Climate & Energy College School of Geography, Earth and Atmospheric Sciences The University of Melbourne Parkville VIC Australia; ^3^ Climate Resource Northcote VIC Australia; ^4^ Priestley International Centre for Climate University of Leeds Leeds UK; ^5^ CICERO Center for International Climate Research Oslo Norway; ^6^ Centre for Environmental Policy Imperial College London London UK; ^7^ Grantham Institute for Climate Change and the Environment Imperial College London London UK

**Keywords:** climate change, emulators, IPCC, scenarios, AR5, AR6

## Abstract

The IPCC's scientific assessment of the timing of net‐zero emissions and 2030 emission reduction targets consistent with limiting warming to 1.5°C or 2°C rests on large scenario databases. Updates to this assessment, such as between the IPCC's Special Report on Global Warming of 1.5°C (SR1.5) of warming and the Sixth Assessment Report (AR6), are the result of intertwined, sometimes opaque, factors. Here we isolate one factor: the Earth System Model emulators used to estimate the global warming implications of scenarios. We show that warming projections using AR6‐calibrated emulators are consistent, to within around 0.1°C, with projections made by the emulators used in SR1.5. The consistency is due to two almost compensating changes: the increase in assessed historical warming between SR1.5 (based on AR5) and AR6, and a reduction in projected warming due to improved agreement between the emulators' response to emissions and the assessment to which it is calibrated.

## Key Points

- Emulators used in IPCC Special Report on warming of 1.5°C and Sixth Assessment Report are remarkably consistent, despite their entirely new calibrations
- The consistency is due to two compensating factors: change in assessed historical warming and improvements to emulator calibration methods

## 1 Introduction

To assess the characteristics of scenarios in line with different levels of global warming, emission scenarios are grouped in distinct categories based on their global‐mean temperature outcomes (Rogelj et al., 2011). This practice was followed in both SR1.5 (Rogelj et al., 2018) and the Working Group 3 (WG3) Contribution to AR6. The emissions scenarios are typically generated by Integrated Assessment Models (IAMs, Weyant, 2017), which combine assumptions about future population, economy, climate policy and technology to project internally consistent evolutions of future greenhouse gas and other emissions.

Over 400 scenarios were assessed in SR1.5 (Huppmann et al., 2018), and AR6 WG3 assessed over 1,200 (Riahi et al., 2022). During the IPCC drafting process, global‐mean temperature projections for these scenarios have to be calculated in a matter of weeks, which requires computationally efficient models, also known as Earth System model emulators. The temperature projections are then used to categorize scenarios according to their global warming outcomes (Riahi et al., 2022).

SR1.5 and AR6 came to similar conclusions about the transformations required to limit warming to 1.5°C with no or limited overshoot. SR1.5 concluded that, by 2030, CO_2_ emissions should be reduced by about 45% from 2010 levels (Rogelj et al., 2018). AR6 found reductions of 43% (interquartile range of 34%–60%) from 2019 levels. These conclusions were based on analysis by a large team of researchers and were the result of multiple interacting factors. In this paper, we analyze the changes in the emulators that were used as part of the scenario assessment process.

To understand the changes in the emulators, we first take a step back in time. Before AR5, IAMs self‐reported climate outcomes of scenarios. However, climate system representations vary in complexity, sophistication, and accuracy between IAMs (Harmsen et al., 2015; van Vuuren et al., 2011), so comparing self‐reported climate outcomes from different IAMs can be complex and inaccurate. To eliminate the unnecessary noise that results from the use of an unwieldy set of poorly calibrated climate models, the WG3 Contribution to AR5 initiated a harmonized approach to the climate assessment of IAM scenarios (Clarke et al., 2014). IAM scenarios were assessed with a single calibrated climate model, also referred to as a climate emulator, in a probabilistic setup (Meinshausen et al., 2009, 2011; Rogelj et al., 2012). The probabilistic calibration aims to make the climate response of the emulator reflect the state of climate science knowledge and its surrounding uncertainties as closely as possible.

AR5 used the MAGICC6 model to assess the scenarios submitted to the AR5 scenario database as part of the wider assessment process. The 2018 IPCC Special Report on Global Warming of 1.5°C (SR1.5, Forster et al., 2018; Rogelj et al., 2018) also used MAGICC6, together with a second climate emulator, FaIR1.3 (Millar et al., 2017; Smith et al., 2018). At the time of SR1.5, differences in the temperature projections by these emulators remained unexplained and were instead highlighted as a knowledge gap. This affected the certainty with which the global warming implications of scenarios could be assessed and scenarios could be grouped into 1.5°C compatible or 2°C compatible classes (Rogelj et al., 2018). In SR1.5, the decision was made to stay consistent with AR5. As a result, MAGICC6 was used for classification of scenarios in SR1.5 and information from FaIR 1.3 was used to inform the overall uncertainty assessment (Rogelj et al., 2018).

Scientific efforts and lessons learned since SR1.5 have now closed the gap in understanding differences between emulators. Climate emulator intercomparison exercises have developed protocols, facilitating comparisons and analysis of differences between emulators and their calibrations (Nicholls et al., 2021; Nicholls & Lewis, 2021). These advances were applied as part of the AR6 physical science assessment (WGI), where a cross‐chapter activity calibrated and vetted four emulators using a wide range of assessed climate system characteristics. This activity ensured that the probabilistic parameterisations of the emulators closely matched AR6 findings related to equilibrium climate sensitivity (ECS), transient climate response (TCR), transient climate response to emissions, ocean heat uptake, historical temperature observations and the assessed projected global‐mean temperatures under various ScenarioMIP scenarios (O’Neill et al., 2016; Tebaldi et al., 2021).

Comparing this set of AR6‐calibrated climate emulators with previous setups allows us to explore how advances in our understanding of the physical climate system affect which emissions pathways are consistent with holding warming below 1.5°C compared to preindustrial levels. The changes in emulators are the result of both model development and developments in calibration processes. In general, we consider both these effects together and simply refer to them under the umbrella of emulator updates. However, in Section 5.1 we separate them out to provide greater insights into the underlying causes of differences.

Given the widespread use of these emulators in the literature, the analysis is also useful for teams who wish to anticipate and understand the changes when updating from the SR1.5 to the AR6 emulators. Throughout this paper we focus on the difference between MAGICC6, which was used for scenario categorization in SR1.5, and MAGICCv7.5.3, which is used for scenario categorization in AR6 WG3. The differences with FaIR1.3 and FaIRv1.6.2, used in SR1.5 and AR6, respectively, are discussed where appropriate but are not examined in the same detail.

## 2 Terminology

Where possible, we simplify the emulator labels by referring to them only by their names and version number (e.g., MAGICC6, FaIRv1.6.2). However, emulator behavior is the result of the emulator structure (captured by its name and version) and how it has been calibrated that is, both components are required to uniquely identify how the emulator was run. Ignoring the importance of calibration has caused considerable confusion in the past when discussing emulators (see e.g., discussion in Section 5 of Leach et al., 2021). To avoid ambiguity, we provide a breakdown of the model versions, calibrations, key characteristics and their purpose in this study (Table S1 in Supporting Information S1).

## 3 Materials and Methods

To compare our emulators, we run a series of scenarios. These scenarios are defined in terms of anthropogenic emissions between 2010 and 2100 that is, they focus on future emissions, with a small overlap with the past which is used for harmonization with historical emissions and IAM calibration.

We use the 368 scenarios underlying Table 2.4 in SR1.5, a subset of the SR1.5 scenario database's complete set of more than 400 scenarios (Huppmann et al., 2019; Rogelj et al., 2018). These scenarios were collated from the available socioeconomic literature by the authors of the SR1.5 report (via an open call to the emissions projection community). As a result, the scenarios come from a variety of IAMs (Rogelj et al., 2018). We focus on this subset as it formed the basis of many of SR1.5's top‐level statements and excludes scenarios that have greenhouse gas emissions that were deemed unrealistic at the time of SR1.5 or bias the full set because of strong similarity (Rogelj et al., 2018). For these 368 scenarios, we reassess their climate outcomes with the newly AR6‐calibrated emulators and reapply the scenario classification rules from SR1.5. We do not use any scenarios from the AR6 database. Consequently, we know that differences in emulator output are only due to changes in the calibration of the climate emulators and associated changes in our physical science understanding, not because of a change in the scenarios themselves (changes in the scenario database between SR1.5 and AR6 are explored elsewhere, e.g, (Kikstra et al., 2022; Riahi et al., 2022).

We re‐run the SR1.5 scenarios with the AR6‐calibrated emulators using the WG3 climate assessment pipeline (Kikstra et al., 2022). The pipeline is built on three key tools: Aneris for harmonizing the emissions timeseries to historical emissions (Gidden et al., 2018, 2022), Silicone for infilling emissions species not natively reported by the IAMs (Lamboll et al., 2020), and OpenSCM‐Runner for running the climate models (Nicholls et al., 2020).

The MAGICCv7.5.3 and FaIRv1.6.2 AR6 setups are documented in Forster et al. (2021). For the SR1.5 emulators, we use output from the SR1.5 database (Huppmann et al., 2018) without modification.

## 4 Results

### 4.1 Scenario Categorization

**Figure 1:**
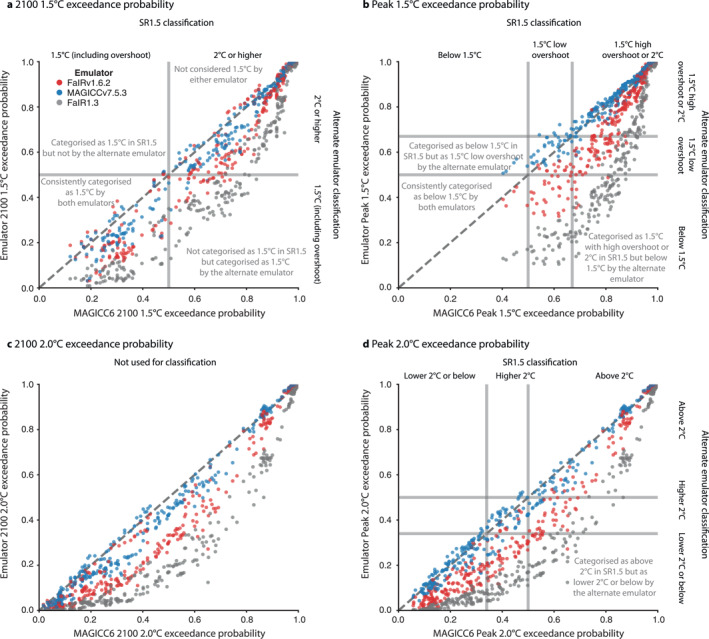
The classification‐relevant exceedance probabilities of Special Report on Global Warming of 1.5°C (SR1.5) scenarios are similar when re‐assessed with MAGICCv7.5.3, slightly lower with FaIRv1.6.2 and lower with FaIR1.3. (a) 1.5°C exceedance probabilities in 2100 from MAGICCv7.5.3 (blue dots), FaIRv1.6.2 (red dots) and FaIR1.3 (gray dots) compared to the data used for SR1.5 categorization that is, MAGICC6. (b) As in panel a, but for peak warming. (c) As in panel a, but for 2°C warming. (d) As in panel a, but for 2°C peak warming. The vertical and horizontal lines delineate the scenario classifications. To aid comparisons, dashed diagonal lines show the 1:1 line (points below the diagonal indicate higher outcomes with MAGICC6 than with the other emulators).

We find that the key outputs used for categorization are broadly consistent between MAGICC6 and MAGICCv7.5.3 (Figure 1). Differences in peak 1.5°C exceedance probability are limited to 0.7% in the median across all the scenarios (5%–95% range across scenarios of −3.5%–4.9%), 0.0% (−9.1%–3.4%) for peak 2.0°C exceedance probability and 0.0% (−11.1%–2.5%) for 2100 1.5°C exceedance probability (Figure S1 in Supporting Information S1). The median difference in median peak warming (across the scenarios) is 0.02°C (−0.15°C–0.06°C) and −0.05°C (−0.16°C–0.05°C) for median 2100 warming (Figures S2 and S3 in Supporting Information S1).

These differences are smaller than the usually applied rounding precision of 0.1°C and natural variability. They demonstrate a remarkable consistency between the SR1.5 and AR6 emulators. For example, AR6 reports assessed temperature projections to the nearest tenth of a degree (Lee et al., 2021). The reason for this choice is the scientific uncertainties that must be considered when making long‐term projections, such as the historical anthropogenic warming uncertainty of 0.8–1.3°C (likely range for 2000–2019 relative to 1850–1900, Eyring et al., 2021), the contribution of internal variability of about 0.15°C for a 20‐year average (5%–95% range, Lee et al., 2021) or uncertainty in the zero emissions commitment (Jones et al., 2019; MacDougall et al., 2020) of about 15% of total warming (1*σ* range, Lee et al., 2021). The contribution of internal variability is key to keep in mind: our climate model emulators only model the externally forced warming response, almost entirely human driven with a small (approximately 1%) contribution from the solar cycle, and natural variations around this are not included in the assessment of warming performed here.

Using MAGICC6, 42 scenarios were classified as 1.5°C with no or low overshoot, 36 were classified as 1.5°C with high overshoot and 54 were classified as lower 2°C (Table 1). Using MAGICCv7.5.3, 41 scenarios are classified as 1.5°C with no or low overshoot, 38 are classified as 1.5°C with high overshoot and 64 are classified as lower 2°C.

**Table 1:** Classification Rules for Scenarios From the IPCC Special Report on Global Warming of 1.5°C (SR1.5) (Only Scenarios Included in SR1.5 Table 2.4, Adapted From Rogelj et al., 2018), Classification of Scenarios in SR1.5 and Classification Based on AR6 Emulators

Class name	Classification rule (P (1.5°C) is the probability that temperatures exceed 1.5°C)	Number of scenarios in SR1.5 Table 2.4	Number of scenarios with other SR1.5 emulator	Number of scenarios with AR6 emulator	
*Emulator*		*MAGICC6*	*FaIR1.3*	*MAGICCv7.5.3*	*FaIRv1.6.2*
Below 1.5°C	0.34 < P (1.5°C) ≤ 0.5	5	127	0	36
1.5°C low‐overshoot	0.5 < P (1.5°C) ≤ 0.67 AND P (1.5°C in 2100) ≤ 0.5	37	22	41	42
1.5°C no or low overshoot	Combination of two categories above that is, P (1.5°C) ≤ 0.67 AND P (1.5°C in 2100) ≤ 0.5	42	149	41	78
1.5°C high‐overshoot	0.67 < P (1.5°C) AND P (1.5°C in 2100) ≤ 0.5	36	1	38	19
Lower 2°C	P (2°C) ≤ 0.34 AND P (1.5°C in 2100) > 0.5	54	76	64	92
Higher 2°C	0.34 < P (2°C) ≤ 0.5 AND P (1.5°C in 2100) > 0.5	54	13	52	36
Above 2°C	P (2°C) > 0.5	182	128	173	143

Using FaIRv1.6.2 and especially FaIR1.3, more scenarios are classified in these low categories due to cooler projections (Table 1). With FaIRv1.6.2, 78 scenarios are assessed as 1.5°C with no or low overshoot, 19 are classified as 1.5°C with high overshoot and 92 are classified as lower 2°C. The lower projections from FaIRv1.6.2 are the result of a slightly lower TCR (Forster et al., 2021; Smith et al., 2021) and lower projections of atmospheric CO_2_ and CH_4_ concentrations (a topic we return to in Section 5.3). If FaIR1.3 had been chosen for the classification of scenarios at the time of SR1.5, a total of 149 scenarios would have been classified as 1.5°C with low or no overshoot, 1 would have been classified as 1.5°C with high overshoot and 76 would have been classified as lower 2°C.

We see the broad consistency between MAGICC6's and MAGICCv7.5.3's projections reflected in the similarity of the scenario classification. The only case where this isn't true is if we draw a distinction between 1.5°C no overshoot and 1.5°C low overshoot scenarios (where 5 scenarios are classified as no overshoot with MAGICC6 while no scenarios are classified as no overshoot using MAGICCv7.5.3, Figure 1). However, making such a distinction means that scenarios in the “1.5°C with low overshoot” category must have a peak 1.5°C exceedance probability between 50% and 67% (a range of approximately 0.12°C in terms of median warming, Figure S4 in Supporting Information S1). Across all scenarios, the changes in 1.5°C exceedance probabilities are much less than this. However, in very strong mitigation scenarios, changes of approximately 10% are seen, which is enough to cause all the “1.5°C no overshoot” scenarios to be reclassified as “1.5°C low overshoot” scenarios when using MAGICCv7.5.3.

### 4.2 Temperature Threshold Crossing Times

**Figure 2:**
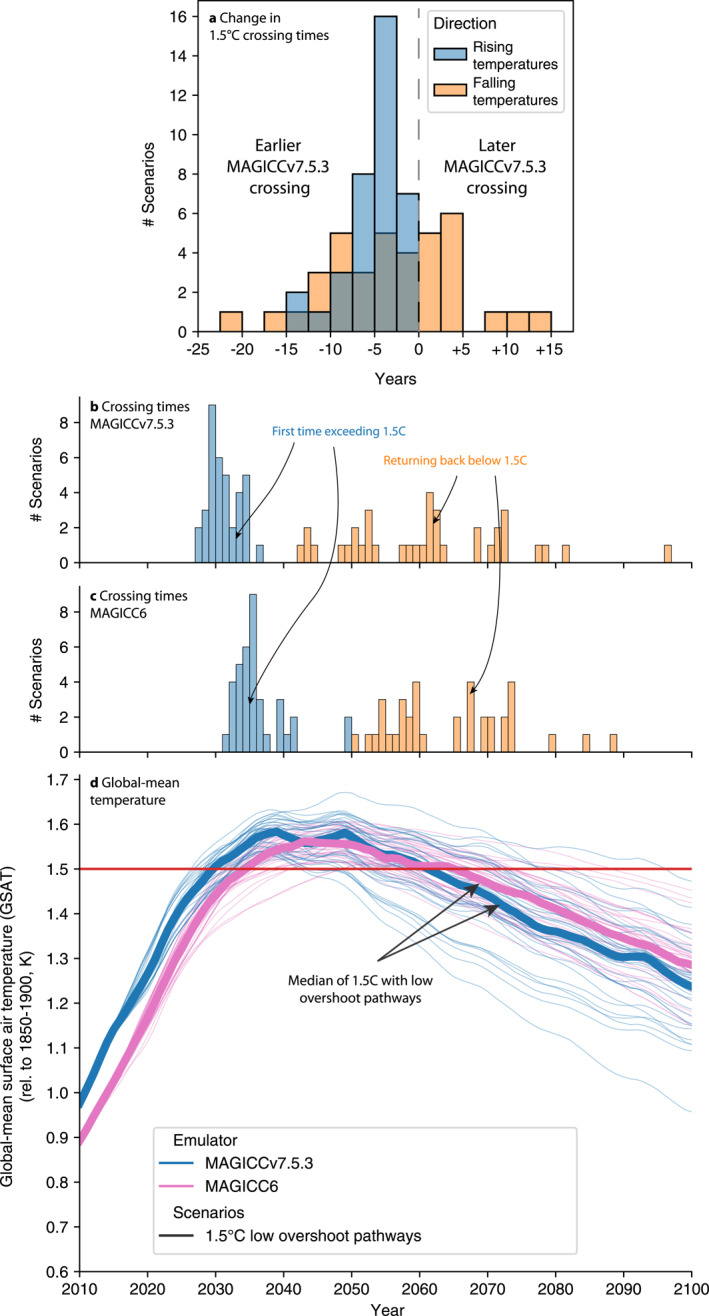
Change in time at which 1.5°CC warming is first crossed and then returned below in scenarios which were classified as 1.5°CC with low overshoot in Special Report on Global Warming of 1.5°C (SR1.5). (a) Crossing times based on MAGICCv7.5.3 relative to the crossing times based on the SR1.5 data (MAGICC6). (b) Crossing times based on MAGICCv7.5.3. (c) Crossing times based on the SR1.5 data (MAGICC6). (d) Timeseries of temperature evolution in the considered pathways.

Alongside the changes in categories, we also consider the change in the point in time when overshoot scenarios cross and return below the 1.5°C threshold (Figure 2). We find that, while scenarios cross the 1.5°C threshold 4 years earlier (in the median) using MAGICCv7.5.3 compared to MAGICC6, many scenarios also return below 1.5°C sooner than previously thought. However, there is quite some uncertainty in the change in the year in which temperatures return below 1.5°C, with the median being a 4 years earlier return and a 5%–95% range of 19 years earlier to 12 years later. The range reflects the fact that small changes in the rate of cooling lead to large changes in crossing times (a result of the geometry of determining the point at which two nearly parallel lines, the 1.5°C limit and the declining temperatures, cross). In addition, both the uncertainty in the climate system's response to net zero or net negative CO_2_ emissions and the wide range of non‐CO_2_ emissions pathways (specifically after net zero CO_2_) in the SR1.5 database contribute to the uncertainty as to when exactly temperature will return back below the 1.5°C limit if temporarily overshot. As a result, it may be more robust to discuss the decade of peak warming and the decadal rate of temperature reduction thereafter rather than the year in which temperatures return back below 1.5°C (Rogelj et al., 2019).

## 5 Discussion

### 5.1 Causes of Categorization Changes

We find two key causes for changes in the IPCC categorization: changes in the historical temperature assessment and other changes in the physical science assessment, which includes the ability of calibrated emulators to reflect that science. The upwards revision of the historical warming in AR6 meant that the best‐estimate for 1986–2005 relative to 1850–1900 was 0.69°C, compared to 0.61°C in AR5 (Gulev et al., 2021). Similarly, for 2003–2012 relative to 1850–1900, AR6's best‐estimate warming was 0.90°C, compared to 0.78°C in AR5. These increases are approximately 0.1°C, or around 15% in terms of 1.5°C exceedance probabilities (Figure S4 in Supporting Information S1).

To disentangle the multiple updates between MAGICC6 and MAGICCv7.5.3, we use three different model setups (Table S1 in Supporting Information S1). On top of MAGICC6 and MAGICCv7.5.3, we add another set of runs with MAGICCv7.5.3. These runs are performed with a calibration that is representative of AR5 science, labeled “AR5‐like MAGICCv7.5.3.” Specifically, we use MAGICCv7.5.3's RCMIP Phase 2 HadCRUT4.6.0.0 calibration (Nicholls et al., 2021) and the AR5 recent past warming estimate of 0.61°C for 1986–2005 relative to 1850–1900.

First, we diagnose the difference between MAGICC6 and MAGICC7, excluding the effect of calibrating to AR6's historical temperature assessment. To do this, we compare results using MAGICC6 and AR5‐like MAGICCv7.5.3.

**Figure 3:**
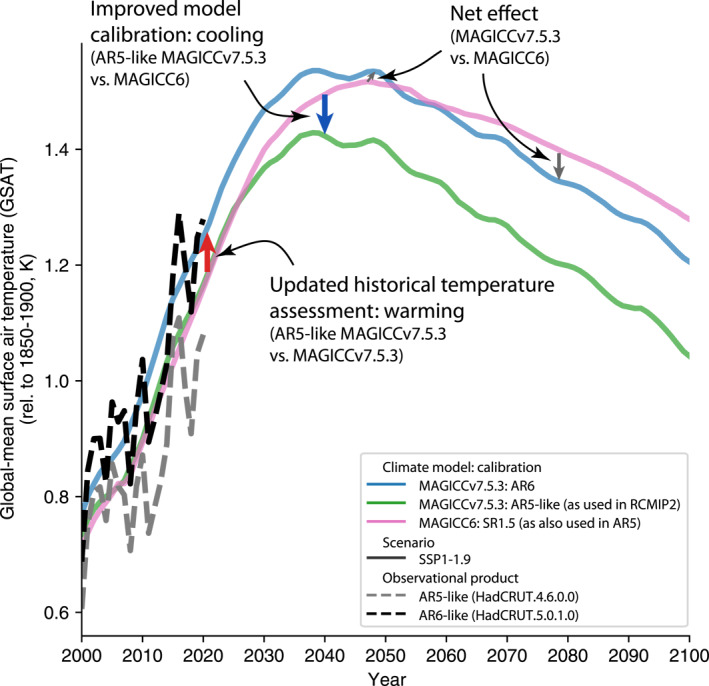
Contributions to changes in temperature projections, illustrated using the SSP1‐1.9 scenario. We compare MAGICC6 as used in Special Report on Global Warming of 1.5°C (SR1.5) (pink line), “AR5‐like” MAGICCv.7.5.3 (green line) and MAGICCv7.5.3 as used in AR6 (blue line). For comparison, we also plot HadCRUT4.6.0.0 (gray dashed line) and HadCRUT5.0.1.0 (black dashed line). HadCRUT4.6.0.0 is used as a proxy for the AR5 historical temperature assessment (which MAGICC6 and AR5‐like MAGICCv.7.5.3 are calibrated to) while HadCRUT5.0.1.0 is used as a proxy for the AR6 historical temperature assessment (which MAGICCv7.5.3 is calibrated to).

AR5‐like MAGICCv7.5.3 projects median peak warming that is 0.13°C less (5%–95% range across scenarios of 0.25°C less to 0.06°C less) than MAGICC6 (Figure 3 and Figures S5 and S6 in Supporting Information S1).

The difference can arise from changes in any of the steps (specifically parameterisations thereof) along the cause‐effect chain from emissions to atmospheric concentrations to effective radiative forcing to warming. We first observe that MAGICC6 generally has lower effective radiative forcing than AR5‐like MAGICCv7.5.3 (Figure S7 in Supporting Information S1, Text S1 in Supporting Information S1). Therefore, differences in the parameterisations that link emissions and effective radiative forcing are not the reason for higher warming projections when using MAGICC6.

Given that effective radiative forcings do not explain the change, we instead focus on the parameterization linking effective radiative forcing and warming. A key measure of this is the TCR. In MAGICC, TCR is not a model parameter, but an emergent property that is influenced by multiple parameters that control ocean heat uptake and climate feedbacks. In AR5, the assessment was a likely range from 1 to 2.5°C (with no explicit central assessment). As AR5‐like MAGICCv7.5.3 is in line with, it not a bit higher than, the AR5 TCR assessment (Nicholls et al., 2021), we conclude that the calibration of MAGICC6 used in SR1.5 had a TCR which was higher than assessed ranges available at the time (as also suggested by Leach et al., 2018).

In other words, updating from MAGICC6 to a setup more directly calibrated to AR5 would likely cause a drop in the projected temperatures. The major driver for this change direct calibration against key climate system properties such as ECS, TCR, and TCR, with other effects playing only a minor role.

Next, we can re‐consider the overall change that is, the difference in warming projections between MAGICC6 and MAGICCv7.5.3 (Figure S8 in Supporting Information S1). The overall change in projections between MAGICCv7.5.3 and MAGICC6 includes both the warming from changes in the IPCC assessment of historically observed warming and the cooling from other forcing and feedback related changes, which manifest in a lower TCR in MAGICCv7.5.3 version compared to MAGICC6. The two contributions (historical warming and other effects) approximately cancel, leading to changes in exceedance probabilities of around 10% as discussed previously.

### 5.2 Implications for Mitigation

**Figure 4:**
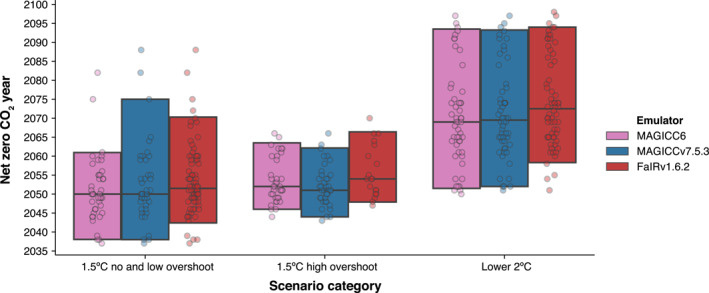
Sensitivity of net zero CO_2_ year in different categories to emulator choice. For each category (*x*‐axis), we show the distribution (black line shows median, box shows 5%–95% range and dots show individual scenarios) of net zero CO_2_ year based on either the SR1.5 classification emulator (MAGICC6), MAGICCv7.5.3 or FaIRv1.6.2 (both as used in AR6). For the number of scenarios in each distribution, see Table 1.

The relatively small differences in climate projections lead to small changes in key mitigation milestones describing scenario categories, such as the year of net zero CO_2_ (Figure 4) or 2030 emissions reductions. Using MAGICC6, no and low overshoot 1.5°C scenarios had a net zero CO_2_ year of 2050 (2038–2061 5%–95% range). Similarly, MAGICCv7.5.3 implies a net zero CO_2_ year of 2050 (2038–2075) and FaIRv1.6.2 implies a net zero CO_2_ year of 2052 (2042–2070).

The importance of these changes for policy and economic transition is a separate question, but they may not be seen as zero in all contexts (e.g., the difference in the 95th percentile is 14 years). These differences in mitigation milestones arise even though climate science has remained remarkably consistent (differences of 0.05°C in the median). A key point from SR1.5 also remains relevant, “because of numerous geophysical uncertainties and model dependencies […] absolute temperature characteristics of the various pathway categories are more difficult to distinguish than relative features” (Rogelj et al., 2018). The fact that our classifications rely on absolute temperatures, in which we have lower confidence, raises the question of whether there are ways to analyze mitigation pathways that rely on the relative differences where we have more confidence.

Another point which is not always immediately obvious is that the connection between changes in physical climate assessment and emissions milestones for scenario categories is not one‐to‐one. For example, the year in which net zero CO_2_ is reached in 1.5°C with low overshoot scenarios and 1.5°C with high overshoot scenarios is similar despite their (by definition) different climate outcomes (Figure 4). The key reason is that the SR1.5 scenario database can be described as an ensemble of opportunity (Huppmann et al., 2018; Rogelj et al., 2011; Tebaldi & Knutti, 2007) and is not a systematic sample of the underlying scenario space (Fujimori et al., 2019).

### 5.3 Emissions‐Driven Uncertainty

The MAGICC and FaIR emulators show improved agreement in AR6 compared to SR1.5. This is particularly so in experiments where concentrations of greenhouse gases are prescribed to the models, where the emulators' median warming projections agree to within 0.05°C under the SSP1‐1.9 and SSP1‐2.6 scenarios (Forster et al., 2021; Smith et al., 2021). These concentration‐driven experiments are directly comparable to both the Working Group 1 (WG1) temperature assessment (Eyring et al., 2021; Gulev et al., 2021) and CMIP ScenarioMIP (Eyring et al., 2016; O’Neill et al., 2016) experiments, both of which are based on large scientific efforts.

However, the agreement between emulators is reduced once we consider experiments where emissions of greenhouse gases are prescribed to the models, rather than concentrations. The switch to emissions‐driven experiments introduces uncertainty in greenhouse gas cycles, particularly the carbon and methane cycles (Forster et al., 2021).

In their AR6‐calibrations, MAGICCv7.5.3 projects higher and methane concentrations than FaIRv1.6.2 (Figure S10 in Supporting Information S1). Unfortunately, a lack of validation data for emissions‐driven experiments, particularly in scenarios where emissions are falling or net negative, restricts our ability to derive robust conclusions about which one of the two projections are more likely. The AR6‐calibrated FaIRv1.6.2's airborne fraction is slightly closer to Earth System Model (ESM) experiments (Forster et al., 2021), although this is based on idealized rather than scenario‐based experiments. There are also few ESM experiments to compare with the methane projections and none which are directly comparable.

Another key uncertainty in these emissions‐driven experiments is the zero emissions commitment, which has a range of −0.34°C–0.28°C (for the change in temperature 50 years after CO_2_ emissions compatible with warming of around 2°C cease) across ESMs (Lee et al., 2021), and was assessed by AR6 to be centered around zero and likely (with greater than 66% probability) fall in the ±0.3°C range.

These carbon and methane cycle differences are part of the reason for differences in MAGICCv7.5.3's and FaIRv1.6.2's temperature projections (Figures S11 and S12 in Supporting Information S1). Improvements in reduced complexity carbon and methane cycle representations and their evaluation is a clear area for future research. Nonetheless, the difference in model projections of order 0.1°C is a reasonable representation of our current emissions‐driven uncertainty. It is also worth noting the progress seen since SR1.5, where emulator disagreement was around 0.3°C in the median and largely unexplained.

## 6 Conclusions

When applied to the SR1.5 scenarios database, the projections from the AR6‐calibrated emulators are remarkably close to their predecessors used in SR1.5. From a climate model emulator perspective, the key insights from SR1.5 remain valid and policies enacted based on the key insights from SR1.5 are supported by the latest scientific evidence. For example, reducing CO_2_ emissions by 50% by 2030 and reaching net zero CO_2_ emissions around 2050 will ‐ from a geophysical perspective ‐ more likely than not limit peak warming to around 1.5°C (i.e., with greater than 50% likelihood). Updates to the design of scenarios (Riahi et al., 2021; Rogelj et al., 2019) with stronger reductions early on and slower approaches toward net‐zero might add further insights into how near‐term action can allow for net zero to be reached later, but they do not change the validity of a 2050 net‐zero CO_2_ year as a guide to mitigation action in the next one or two decades given current emission trends.

Our best projection remains that the world is going to see 1.5°C warming by the early 2030s (averaged over a 20‐year period and acknowledging that individual years will exceed 1.5°C beforehand due to natural variability). Thus, while decisive mitigation efforts this decade will be crucial in determining whether we shoot beyond 1.5°C, adaptation actions will have to be taken on the basis of a minimal warming level around 1.5°C.

## Supporting information

Supporting Information S1

## Data Availability

The code and data used to produce the plots is preserved at https://doi.org/10.5281/zenodo.6584385 and developed openly at https://gitlab.com/magicc/nicholls-et-al-2022-emulator-changes.
