# Supplementary material for: Changes in IPCC Scenario Assessment Emulators Between SR1.5 and AR6 Unraveled
Source: Geophys Res Lett. 2022 Oct 18;49(20):e2022GL099788. doi: 10.1029/2022GL099788 (PMC9788315; doi:10.1029/2022GL099788)
Supplement: Supplementary file 1 — Supporting Information S1 [file GRL-49-e2022GL099788-s001.pdf]

Supporting Information for 'Changes in IPCC scenario assessment emulators between SR1.5 and AR6 unravelled'

Z. Nicholls[1,2,3], M. Meinshausen[2,3], J. Lewis[1,2,3], C. J. Smith[1,4], P. M. Forster[4], J. Fuglestvedt[5], J. Rogelj[1,6,7], J. S. Kikstra[1,6,7], K. Riahi[1], E. Byers[1]

[1]International Institute for Applied System Analysis, IIASA, Laxenburg, Austria

[2]Climate & Energy College, School of Geography, Earth and Atmospheric Sciences, The University of Melbourne, Parkville, Victoria, Australia

[3]Climate Resource, Northcote, Victoria, Australia

[4]Priestley International Centre for Climate, University of Leeds, Leeds, United Kingdom

[5]CICERO, Oslo, Norway

[6]Centre for Environmental Policy, Imperial College London, London, United Kingdom

[7]Grantham Institute for Climate Change and the Environment, Imperial College London, United Kingdom

Contents of this file

Corresponding author: Z. Nicholls, Climate & Energy College, School of Geography, Earth and Atmospheric Sciences, The University of Melbourne, Parkville, Victoria, Australia (zebedee.nicholls@climate-energy-college.org)

1. Text S1: Reasons for the higher warming projections in MAGICC6 compared to the projections from AR5-like MAGICCv7.5.3

2. Text S2: Changes in effective radiative forcing by species between MAGICC6 and MAGICCv7.5.3

3. Figure S1: Difference between the emulator output underlying the SR1.5 scenario categorisation and alternate emulator output for exceedance probabilities of 1.5°C and 2°C warming for 2100 and peak

4. Figure S2: Projected median and upper 67% warming projections for SR1.5 scenarios between MAGICC6, MAGICCv7.5.3, FaIRv1.6.2 and FaIR 1.3

5. Figure S3: Difference between the emulator output underlying the SR1.5 scenario categorisation and alternate emulator output for median and 67th-percentile projections for 2100 and peak temperatures

6. Figure S4: Relationship between exceedance probabilities and temperature projections across multiple emulators

7. Figure S5: Difference between the emulator output underlying the SR1.5 scenario categorisation and AR5-like MAGICCv7.5.3 for median and 67th-percentile projections for 2100 and peak temperatures

8. Figure S6: Difference between the emulator output underlying the SR1.5 scenario categorisation and AR5-like MAGICCv7.5.3 for median and 67th-percentile projections for 2100 and peak temperatures shown as histogram rather than scatter plot

9. Figure S7-S8, S12-S15: Comparison of output from the emulator underlying the SR1.5 scenario categorisation (MAGICC6) and MAGICCv7.5.3

10. Figure S9: CO_2 and CH_4 concentration projections from the MAGICCv7.5.3 and FaIRv1.6.2

11. Figure S10: Difference between MAGICCv7.5.3 and FaIRv1.6.2 emulators for median and 67th-percentile projections for 2100 and peak temperatures

12. Figure S11: Difference between MAGICCv7.5.3 and FaIRv1.6.2 emulators for median and 67th-percentile projections for 2100 and peak temperatures shown as a histogram rather than scatter plot

13. Figure S12-S17: Comparison of output from the emulator underlying the SR1.5 scenario categorisation (MAGICC6) and AR5-like MAGICCv7.5.3

14. Figure S18-S21: Comparison of more output from the emulator underlying the SR1.5 scenario categorisation (MAGICC6) and MAGICCv7.5.3

15. Table S1: Emulator model versions, their key characteristics and the main purpose for their use in this study

Text S1.

Here we discuss the reasons for the higher warming projections in MAGICC6 compared to the projections from AR5-like MAGICCv7.5.3 (Supplementary Figure S13). MAGICC6 generally has lower anthropogenic effective radiative forcing than AR5-like MAGICCv7.5.3 (Supplementary Figure S7). Therefore, differences in the parameterisations that link emissions and effective radiative forcing are not the reason for MAGICC6's higher warming projections and we hence conclude that differences in model calibration, particularly transient climate response (TCR), explain the difference instead.

We next discuss some of the changes in effective radiative forcing of different species and why these changes are approximately zero. As the AR5-like calibration of MAGICCv7.5.3 reflects literature which is more recent than AR5, this comparison provides an insight into some, but not all, the changes in effective radiative forcing between AR5 and AR6.

CO_2 effective radiative forcing is again relatively unchanged between the two emulators (Supplementary Figure S14), reflecting the close agreement between the CO_2 ERF estimates used for the RCMIP Phase 2 tuning (Smith et al., 2020) and the AR5 assessment (Myhre et al., 2013). The differences in CO_2 effective radiative forcing are again largely driven by differences in the carbon cycle response (Supplementary Figure S15), with a similar range of differences on either side of the median.

Aerosol effective radiative forcing is more sensitive to changes in aerosol emissions in AR5-like MAGICCv7.5.3 than MAGICC6 (Supplementary Figure S16). These changes arise because the ERF used in RCMIP Phase 2 (Smith et al., 2020) assesses aerosol forcing for 1750-2014 to be -1.01 W m^{-2}, an approximately 10% increase on AR5's 1750-2011 assessment (Myhre et al., 2013) of -0.9 W m^{-2}.

Methane effective radiative forcing increases (Supplementary Figure S17). The increase follows the upwards revision presented in (Etminan et al., 2016, without the inclusion of rapid adjustments, which are not included in AR5-like MAGICCv7.5.3).

Taking all the changes together, we see that CO_2 effective radiative forcing is largely unchanged, aerosol effective radiative forcing is initially more negative before being more positive and methane effective radiative forcing is more positive. In sum, the combination of these and other changes leads to an increase of 0.0 - 0.2 W m^{-2} in anthropogenic

effective radiative forcing. We note again that these changes are scenario dependent (Supplementary Figure S10) and bespoke analysis is required to understand each specific scenario's drivers.

Text S2.

Here we consider the changes in effective radiative forcing of different species and why these changes cancel out to approximately zero change (with a slight skew towards an increase) in total anthropogenic effective radiative forcing between MAGICC6 and MAGICCv7.5.3.

CO_2 effective radiative forcing has a median change of approximately zero (Supplementary Figure S18). This almost zero change is the result of a slight increase in the assessment of CO_2 effective radiative forcing (for a given atmospheric CO_2 concentration) and a decrease in projected atmospheric CO_2 concentrations (Supplementary Figure S19). It is also worth noting that, while the median is unchanged, there is a range of differences due to differing climate-carbon feedbacks (particularly those driven by the different temperature projections).

Aerosol effective radiative forcing is more sensitive to changes in aerosol emissions. As a result, the negative aerosol effective radiative forcing is stronger today but reduced in the future as aerosol emissions drop (differences are calculated as MAGICCv7.5.3 minus MAGICC6 output, Supplementary Figure S20). These changes arise because the AR6 assessment of ERF (Forster et al., 2021) assesses aerosol forcing for 2005-2014 relative to 1750 to be -1.3 W m^{-2}, compared to AR5's assessment (Myhre et al., 2013) for 2011 relative to 1750 of -0.9 W m^{-2}.

Finally, methane effective radiative forcing increases in the short-term before returning to a median difference of zero with a range of -0.075 W m^{-2} to 0.1 W m^{-2} (Supplementary Figure S21). The increase follows the upwards revision presented in Etminan et al. (2016), tempered by AR6's inclusion of rapid adjustments in the assessment of methane effective radiative forcing in line with Smith et al. (2020). The increased sensitivity to methane emissions (via methane concentrations) also leads to a more pronounced reduction in methane effective radiative forcing after its peak.

In summary, CO_2 effective radiative forcing has a median change of around zero with a slight skew towards a decrease, aerosol effective radiative forcing is initially more negative before being more positive and methane effective radiative forcing is initially more positive before being around zero or slightly more negative. In general, the combination of these and other changes lead to the increase in anthropogenic effective radiative forcing. However, the scenario dependence of these changes should once again be noted as individual scenarios might not match well with the overall trends discussed here (Supplementary Figure S9).

References

Etminan, M., Myhre, G., Highwood, E. J., & Shine, K. P. (2016). Radiative forcing of carbon dioxide, methane, and nitrous oxide: A significant revision of the methane radiative forcing [Journal Article]. *Geophysical Research Letters*, *43*(24), 12,614-12,623. Retrieved from http://dx.doi.org/10.1002/2016gl071930 doi: 10.1002/2016gl071930

Forster, P., Storelvmo, T., Armour, K., Collins, W., Dufresne, J. L., Frame, D., ...

Zhang, H. (2021). The earth's energy budget, climate feedbacks, and climate sensitivity [Book Section]. In V. Masson-Delmotte et al. (Eds.), *Climate Change 2021: The Physical Science Basis. Contribution of Working Group I to the Sixth Assessment Report of the Intergovernmental Panel on Climate Change* (chap. 7). Cambridge University Press. Retrieved from https://www.ipcc.ch/report/ar6/wg1/downloads/report/IPCC_AR6_WGI_Chapter_07.pdf

Myhre, G., Shindell, D., Bréon, F. M., Collins, W., Fuglestvedt, J., Huang, J., ... Zhang, H. (2013). Anthropogenic and natural radiative forcing [Book Section]. In T. F. Stocker et al. (Eds.), *Climate change 2013: The physical science basis. contribution of working group i to the fifth assessment report of the intergovernmental panel on climate change* (p. 659–740). Cambridge, United Kingdom and New York, NY, USA: Cambridge University Press. Retrieved from http://www.climatechange2013.orghttp://dx.doi.org/10.1017/CBO9781107415324.018 doi: 10.1017/CBO9781107415324.018

Nicholls, Z., Meinshausen, M., Lewis, J., Corradi, M. R., Dorheim, K., Gasser, T., ... Woodard, D. L. (2021). Reduced complexity model intercomparison project phase 2: Synthesizing earth system knowledge for probabilistic climate projections [Journal Article]. *Earth's Future*, *9*(6), e2020EF001900. Retrieved from https://agupubs.onlinelibrary.wiley.com/doi/abs/10.1029/2020EF001900 doi: https://doi.org/10.1029/2020EF001900

Smith, C. J., Kramer, R. J., Myhre, G., Alterskjær, K., Collins, W., Sima, A., ... Forster, P. M. (2020). Effective radiative forcing and adjustments in cmip6

models [Journal Article]. *Atmospheric Chemistry and Physics*, *20*(16), 9591-9618. Retrieved from https://acp.copernicus.org/articles/20/9591/2020/http://dx.doi.org/10.5194/acp-20-9591-2020 doi: 10.5194/acp-20-9591-2020

do not specify file extension

Table S1: Emulators and their calibrations, key characteristics and main purpose in this study.

**Model version**	**Calibration**	**Key characteristic**	**Purpose in this study**
MAGICC6	SR1.5/AR5	MAGICC6 with its calibration and input data as used to categorise scenarios in the IPCC SR1.5 report and also used in AR5.	Baseline reference against which we express most emulator variations.
MAGICCv7.5.3	RCMIP Phase 2 (i.e. 'AR5-like')	MAGICCv7.5.3 as used in RCMIP Phase 2, calibrated to match scientific understanding approximately in line with knowledge at the time of SR1.5 (for full details, see Nicholls et al. (2021)). This calibration approximately reflects the science of AR5 and SR1.5, but not the science of AR6.	Provides a MAGICC calibration that approximates the projections might have been made in SR1.5, had MAGICC been re-calibrated at the time. Allows us to disentangle changes from model calibration and changes from model versions. This setup's output provides an indication of what MAGICC6 could have been with updated calibration efforts that better matched the physical science at the time of SR1.5. The key difference to MAGICC6 is a slightly lower TCR - more in line with the AR5 TCR assessment.
MAGICCv7.5.3	AR6	MAGICCv7.5.3 calibrated to AR6 (see Cross-chapter Box 7.1 of Forster et al. (2021))	As this setup was used for scenario categorisation in AR6, a comparison with MAGICC6 indicates the full difference in the climate assessment between SR1.5 and AR6. A comparison with 'RCMIP Phase 2 MAGICCv7.5.3' approximates the effect of the physical science update and subsequent calibration from AR5 to AR6 (separate from model development).

Continued on next page

Table S1 – *Continued from previous page*

**Model version**	**Calibration**	**Key characteristic**	**Purpose in this study**
FaIR1.3	SR1.5	The model used for sensitivity exploration (beyond MAGICC6) as part of the IPCC SR1.5 assessment. The differences between SR1.5 MAGICC6 and SR1.5 FaIR 1.3 were largely unexplained at the time.	Demonstrating, for historical context, the relatively large difference that existed between two key emulators at the time of the IPCC SR1.5.
FaIRv1.6.2	AR6	FaIRv1.6.2 calibrated to AR6 (see Cross-chapter Box 7.1 of Forster et al. (2021))	The difference between AR6 MAGICCv7.5.3 and AR6 FaIRv1.6.2 highlights the uncertainty that arises due to emulator representation of AR6 science. Both AR6 MAGICCv7.5.3 and AR6 FaIRv1.6.2 were assessed as representing WG1 well, so we cannot say with confidence if one is more correct. The difference is much reduced compared to the difference between MAGICC6 and FaIR1.3 (where, had time allowed, deeper investigation may have revealed which model was more in line with the science available at the time).

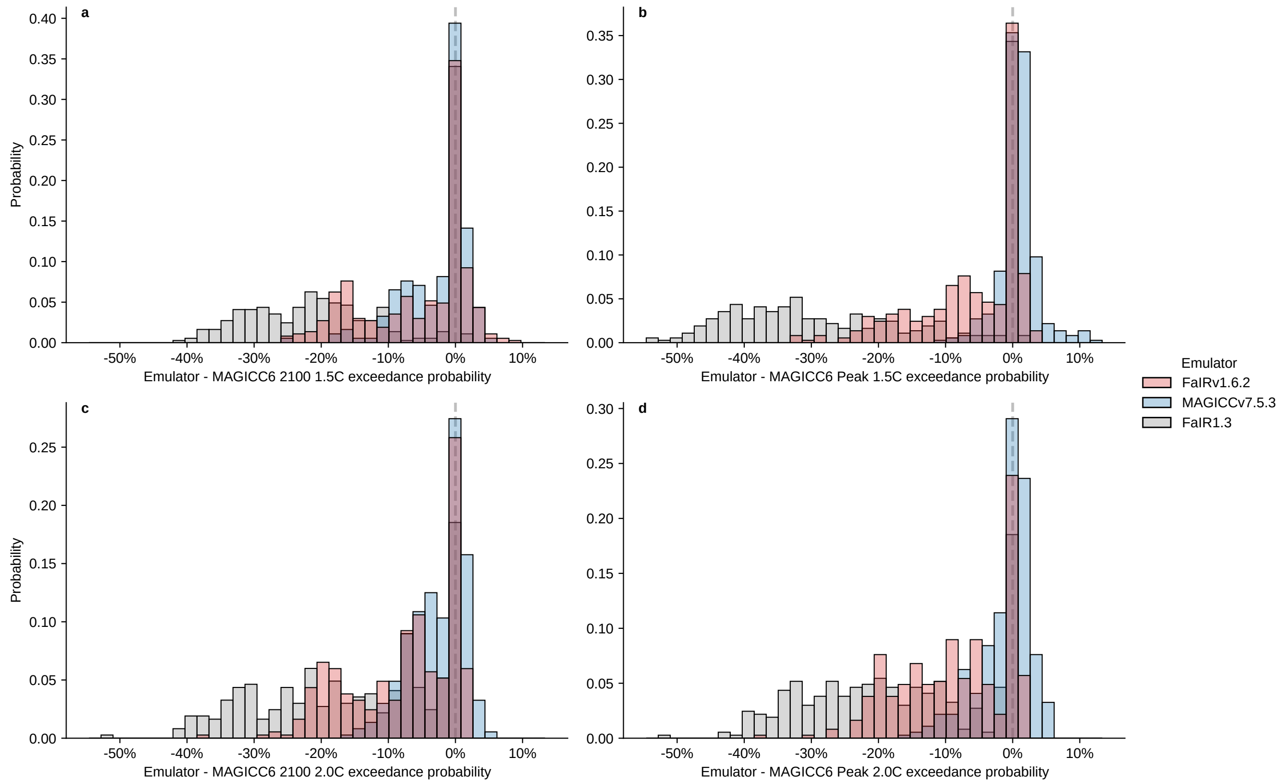

Figure S1. Difference between the emulator output underlying the SR1.5 scenario categorisation and alternate emulator output for exceedance probabilities of 1.5°C (top row) and 2°C warming (bottom row) for 2100 (left column) and peak (i.e. maximum between 2010 and 2100, right column). The data is the same as Figure 1 but has been plotted as a histogram of differences rather than a scatter plot. Scenarios with exceedance probabilities of close to 100% cannot have large changes in exceedance probability (because exceedance probability is capped at 100%), hence the large number of results with no change (zero is marked by the dashed grey line). The difference from MAGICCv7.5.3 emulator is shown in dark blue, from FaIRv1.6.2 is shown in red and from FaIR1.3 is shown in grey.

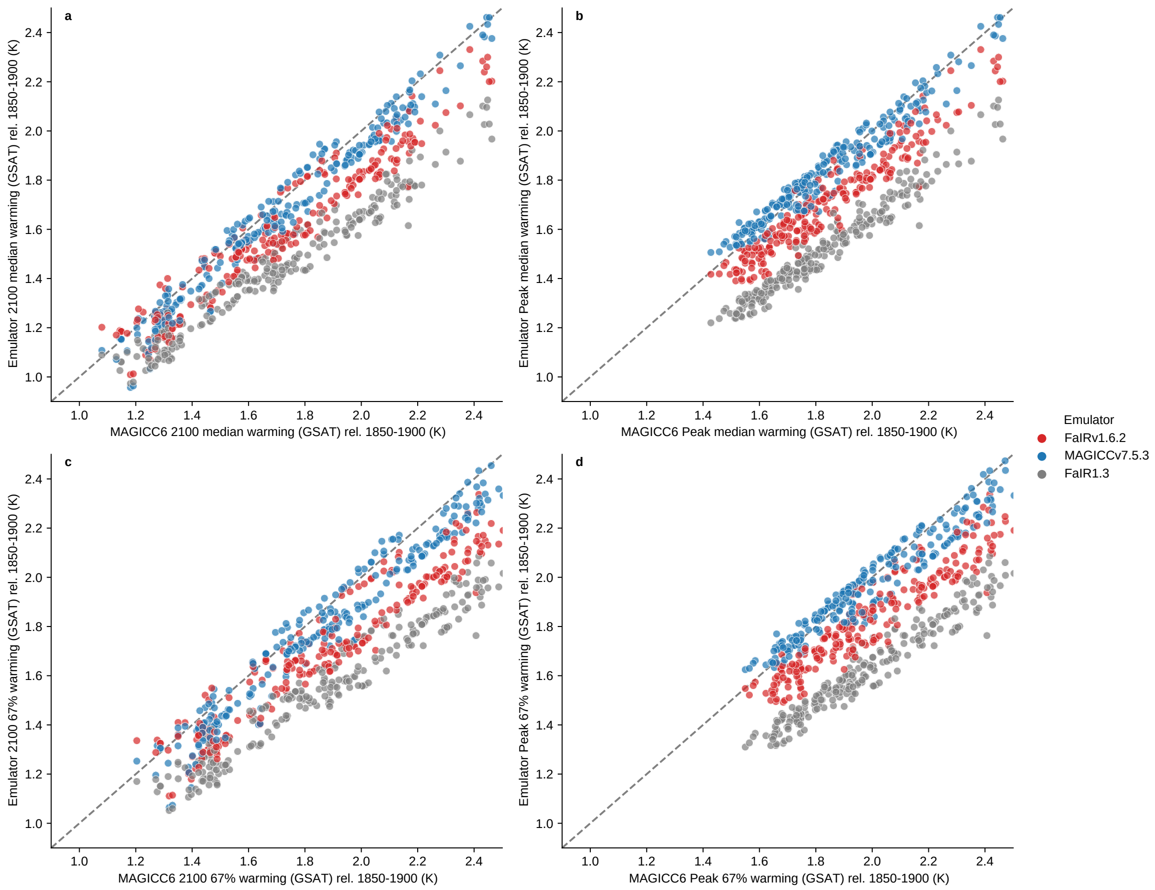

Figure S2. Projected median and upper 67% warming projections for SR1.5 scenarios are very similar between MAGICC6 and MAGICCv7.5.3 (blue dots), slightly higher compared to FaIRv1.6.2 (red dots) or FaIR1.3 (grey dots). Emulator output underlying the SR1.5 scenario categorisation (x-axes) and other emulators (y-axes) for median (top row) and 67th-percentile (bottom row) projections for 2100 (left column) and peak (right column) temperatures. Scenarios on the diagonal (grey dashed line) did not change temperature projections. Scenarios that exhibit lower global-mean temperatures according to the AR6-calibrated emulators are shown below the diagonal. MAGICCv7.5.3 is shown in dark blue, FaIRv1.6.2 is shown in red is and the grey dots indicate FaIR1.3.

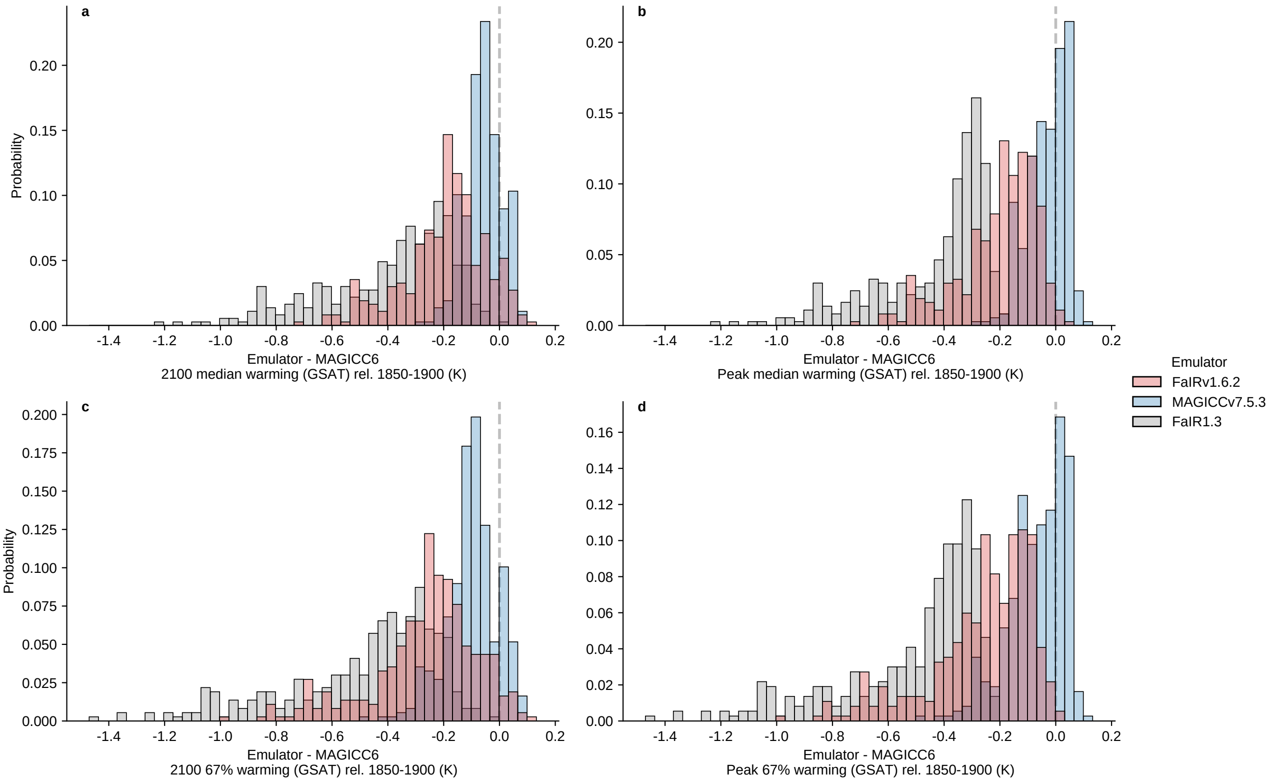

Figure S3. Difference between the emulator output underlying the SR1.5 scenario categorisation and alternate emulator output for median (top row) and 67th-percentile (bottom row) projections for 2100 (left column) and peak (right column) temperatures. The data is the same as Supplementary Figure S2 but has been plotted as a histogram rather than a scatter plot. The difference from MAGICCv7.5.3 is shown in dark blue, from FaIRv1.6.2 is shown in red and from FaIR1.3 is shown in grey.

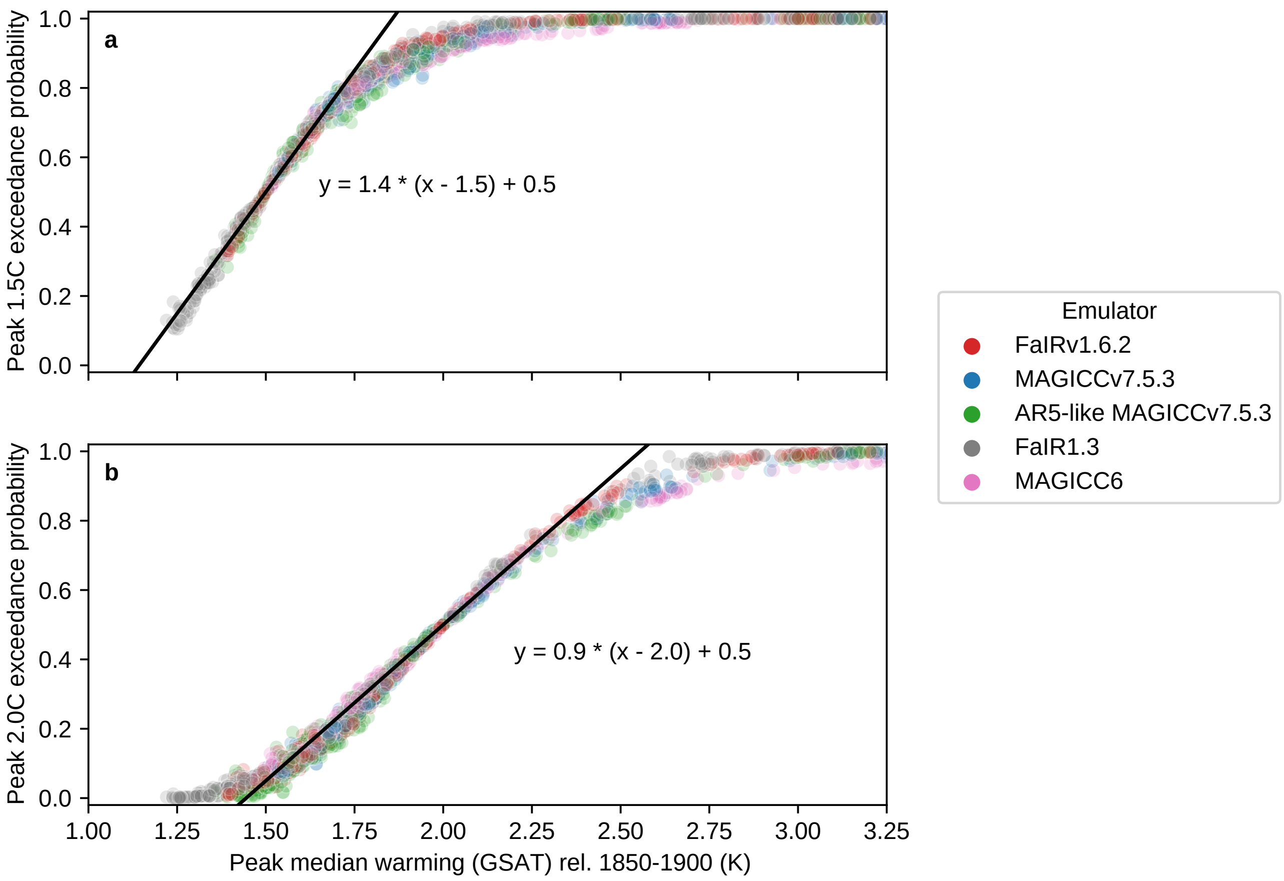

Figure S4. Relationship between exceedance probabilities and temperature projections across multiple emulators. By definition, when the median temperature is 1.5°C (2.0°C), the 1.5°C (2.0°C) exceedance probability is 50%. For every 0.01°C increase in median temperature, 1.5°C exceedance probability increases by around 1.4% and 2.0°C exceedance probability increases by around 0.9%. The difference in gradient is because the uncertainty increases as the median temperature projection increases i.e. we have wider distributions once we get to around 2.0°C warming. The relationship is remarkably consistent across the emulators, with some small variations that become more noticeable as we get into the tails of the distributions i.e. as the median temperature moves away from the exceedance threshold of interest.

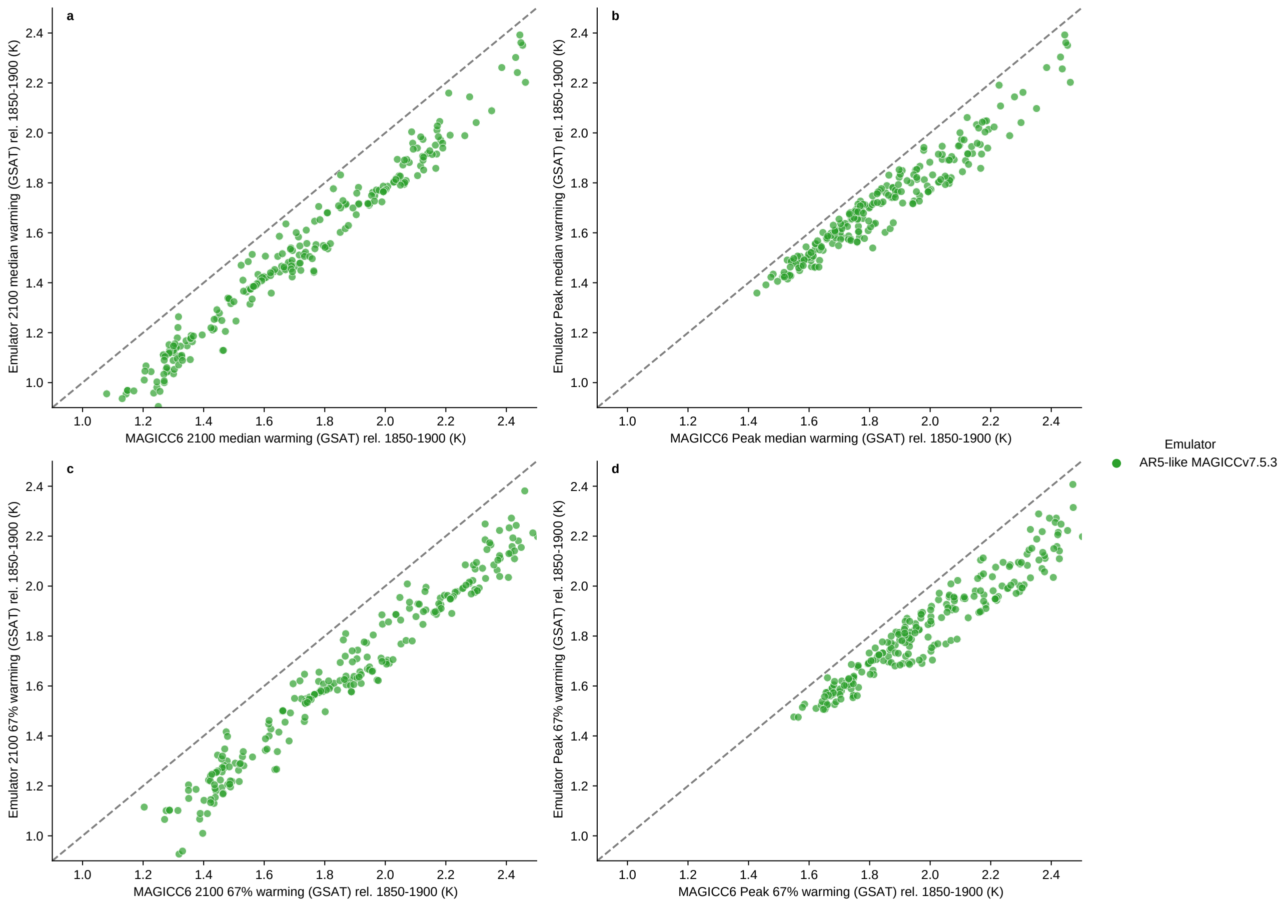

Figure S5. Emulator output underlying the SR1.5 scenario categorisation (x-axes) and AR5-like MAGICCv7.5.3 (y-axes) for median (top row) and 67th-percentile (bottom row) projections for 2100 (left column) and peak (right column) temperatures. If temperature projections hadn't changed for a scenario, that scenario would be shown on the diagonal (grey dashed line). Scenarios that exhibit lower global-mean temperatures according to AR5-like MAGICCv7.5.3 are shown below the diagonal. The difference between AR5-like MAGICCv7.5.3 and MAGICC6 provides an approximate quantification of the change in projections which results from improved emulator calibrations. The figure is the same as Supplementary Figure S2 but for a different combination of models.

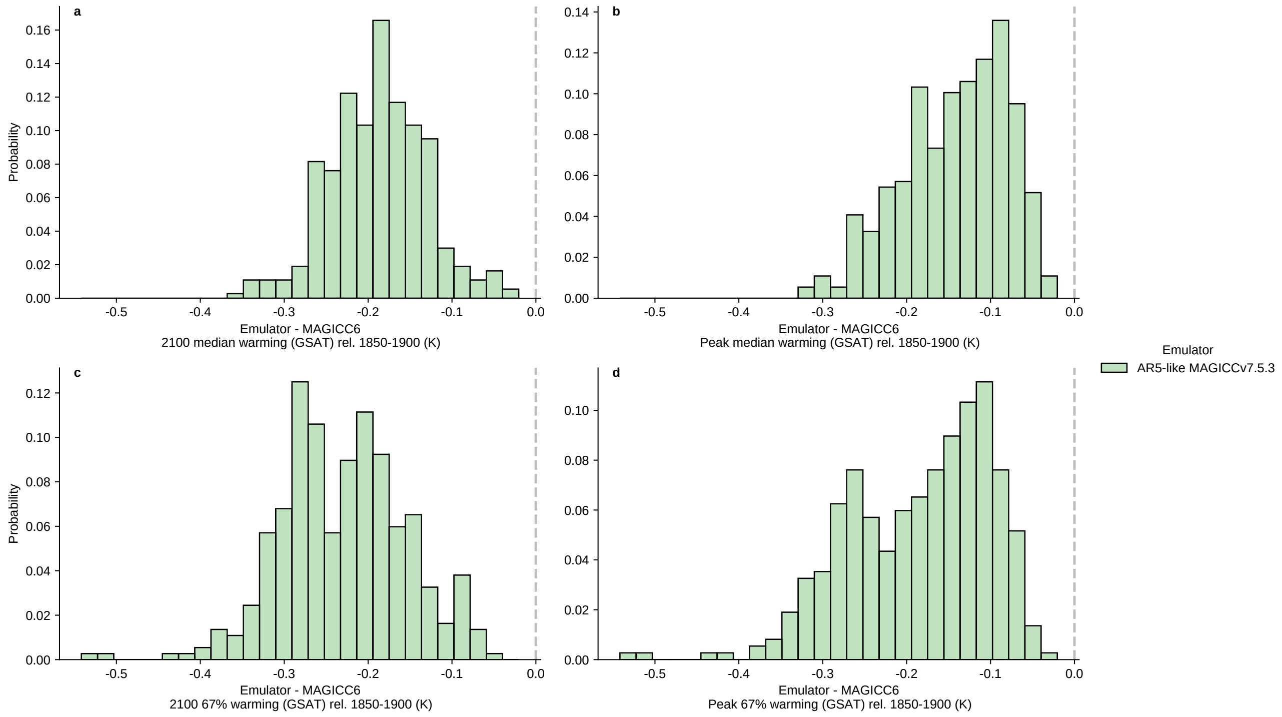

Figure S6. Difference between the emulator output underlying the SR1.5 scenario categorisation and AR5-like MAGICCv7.5.3 for median (top row) and 67th-percentile (bottom row) projections for 2100 (left column) and peak (right column) temperatures. The data is the same as Supplementary Figure S5 but has been plotted as a histogram rather than a scatter plot.

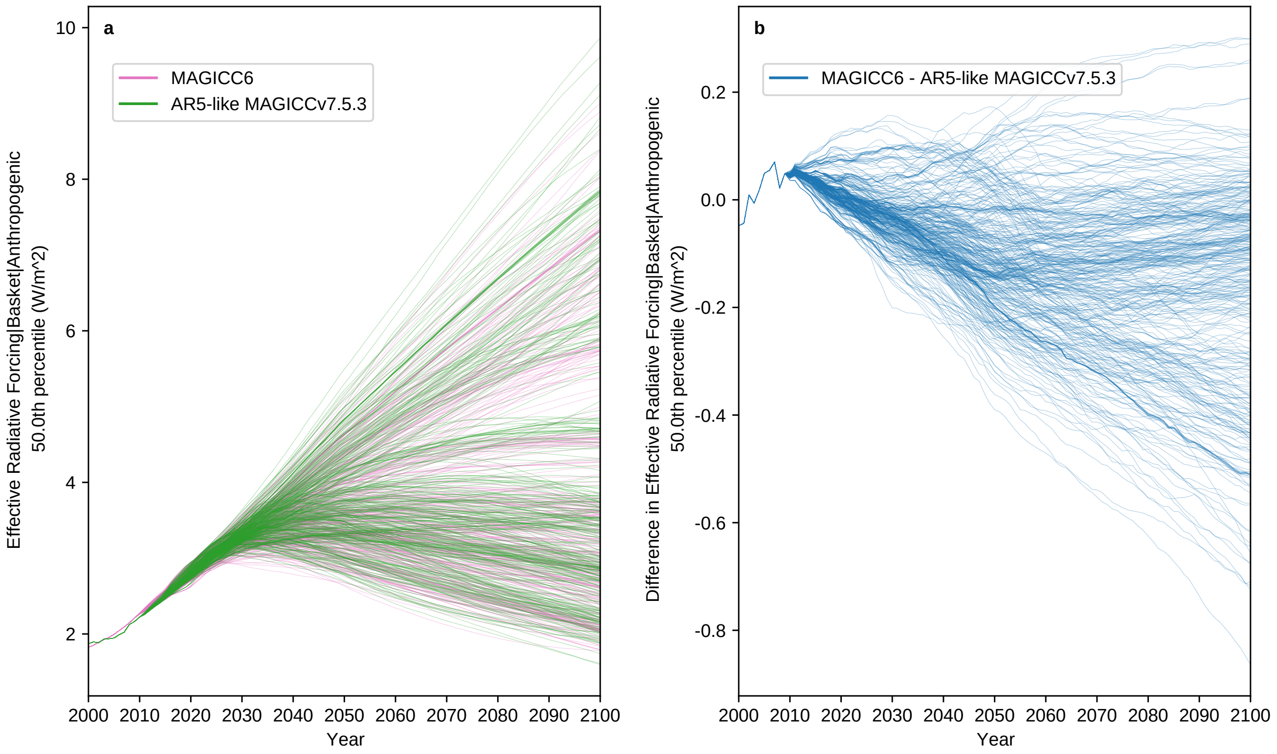

Figure S7. Comparison of output from the emulator underlying the SR1.5 scenario categorisation (MAGICC6) and AR5-like MAGICCv7.5.3. a) Timeseries of median anthropogenic effective radiative forcing. b) Difference (MAGICCv7.5.3 - MAGICC6) in median anthropogenic effective radiative forcing.

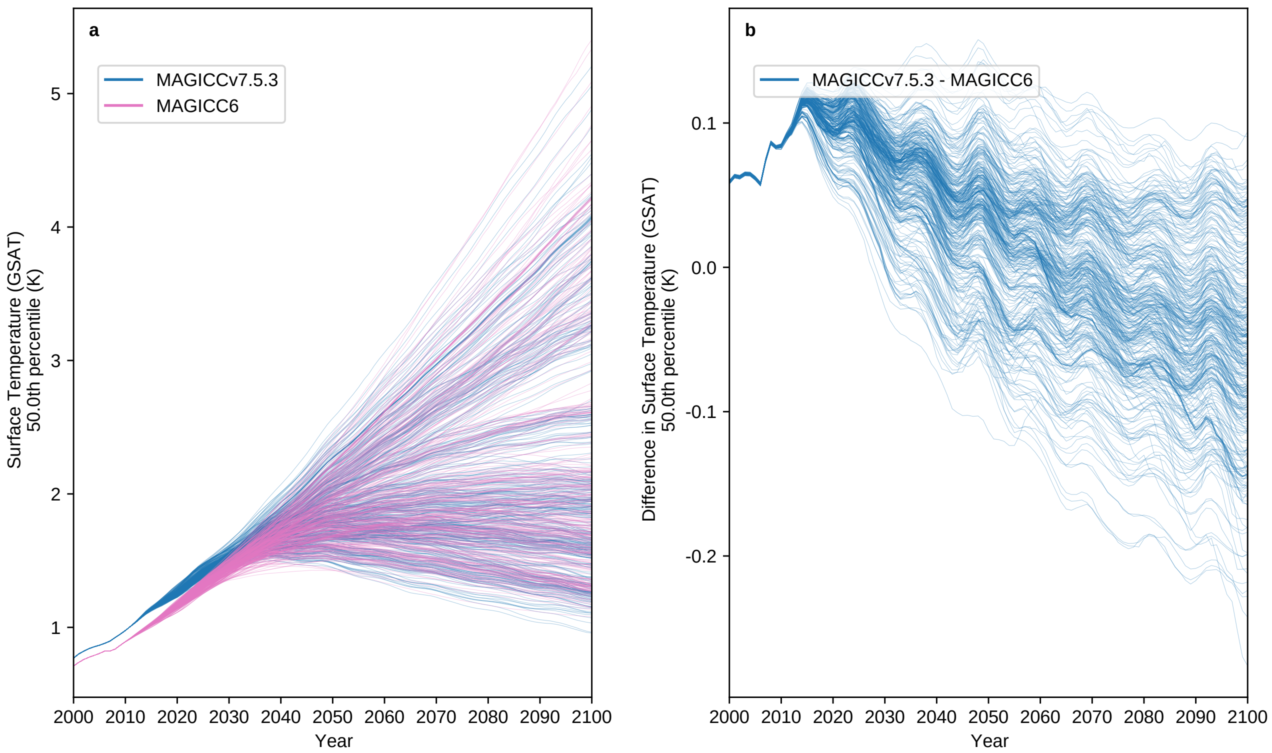

Figure S8. Comparison of output from the emulator underlying the SR1.5 scenario categorisation (MAGICC6) and MAGICCv7.5.3. a) Timeseries of median surface air temperature (GSAT). b) Difference (MAGICCv7.5.3 - MAGICC6) in median surface air temperature (GSAT).

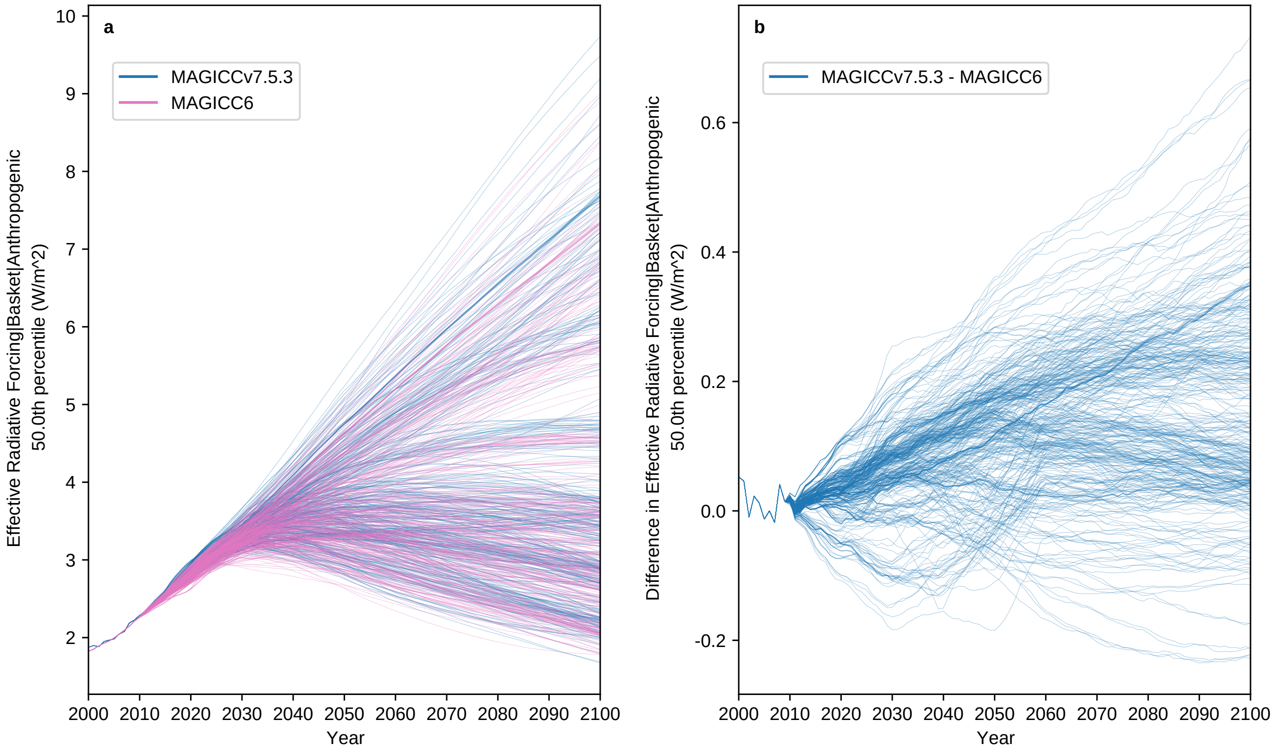

Figure S9. Comparison of output from the emulator underlying the SR1.5 scenario categorisation (MAGICC6) and MAGICCv7.5.3. a) Timeseries of anthropogenic effective radiative forcing. b) Difference (MAGICCv7.5.3 - MAGICC6) in anthropogenic effective radiative forcing.

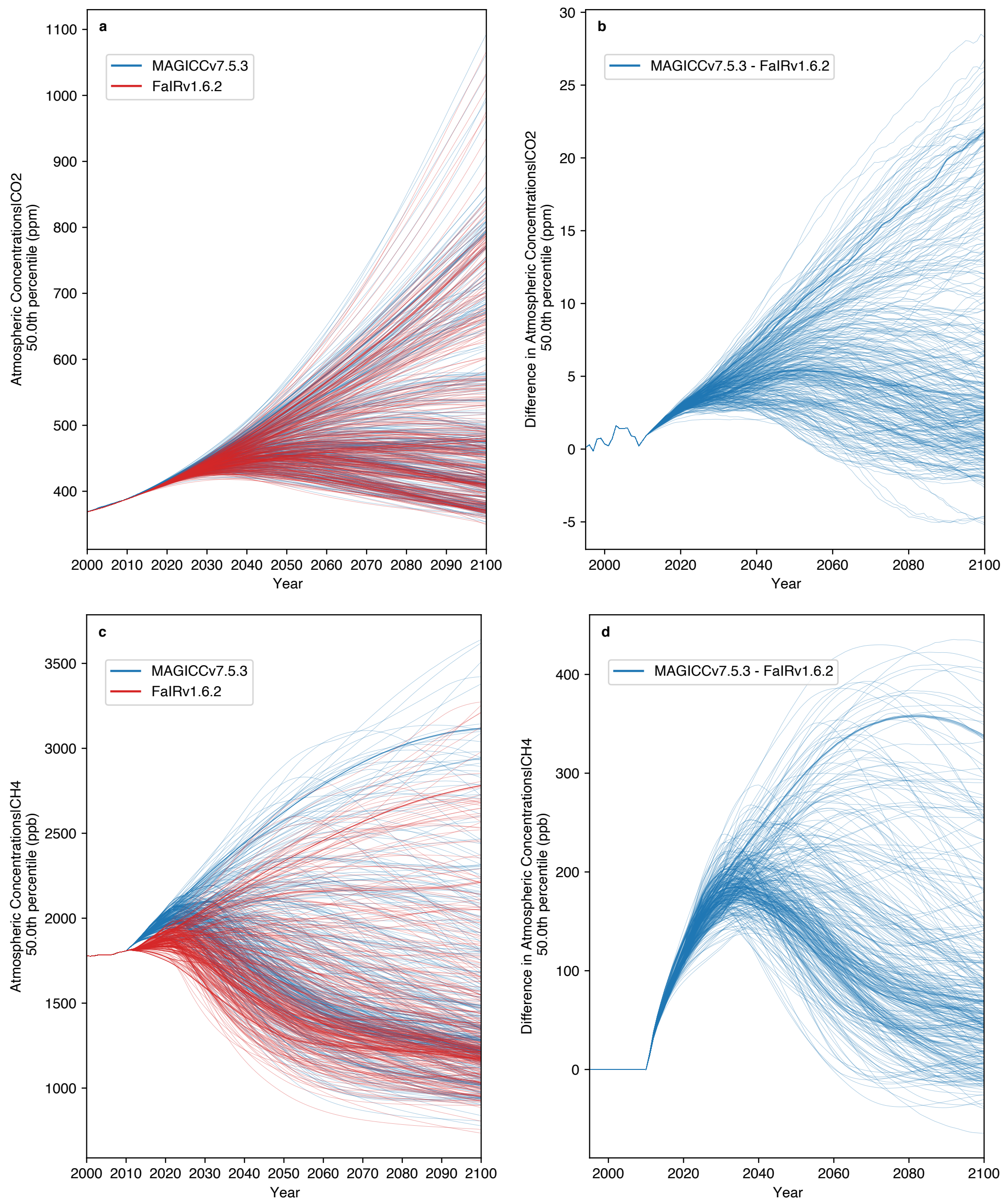

Figure S10. CO_2 (top row) and CH_4 (bottom row) concentration projections from MAGICCv7.5.3 and FaIRv1.6.2. In the first column we show absolute projections from each emulator and in the second column we show the difference between the two emulators. MAGICCv7.5.3 projects higher concentrations (in general) than FaIRv1.6.2 which is part of the reason for differences in emissions-driven runs like those performed in WG3.

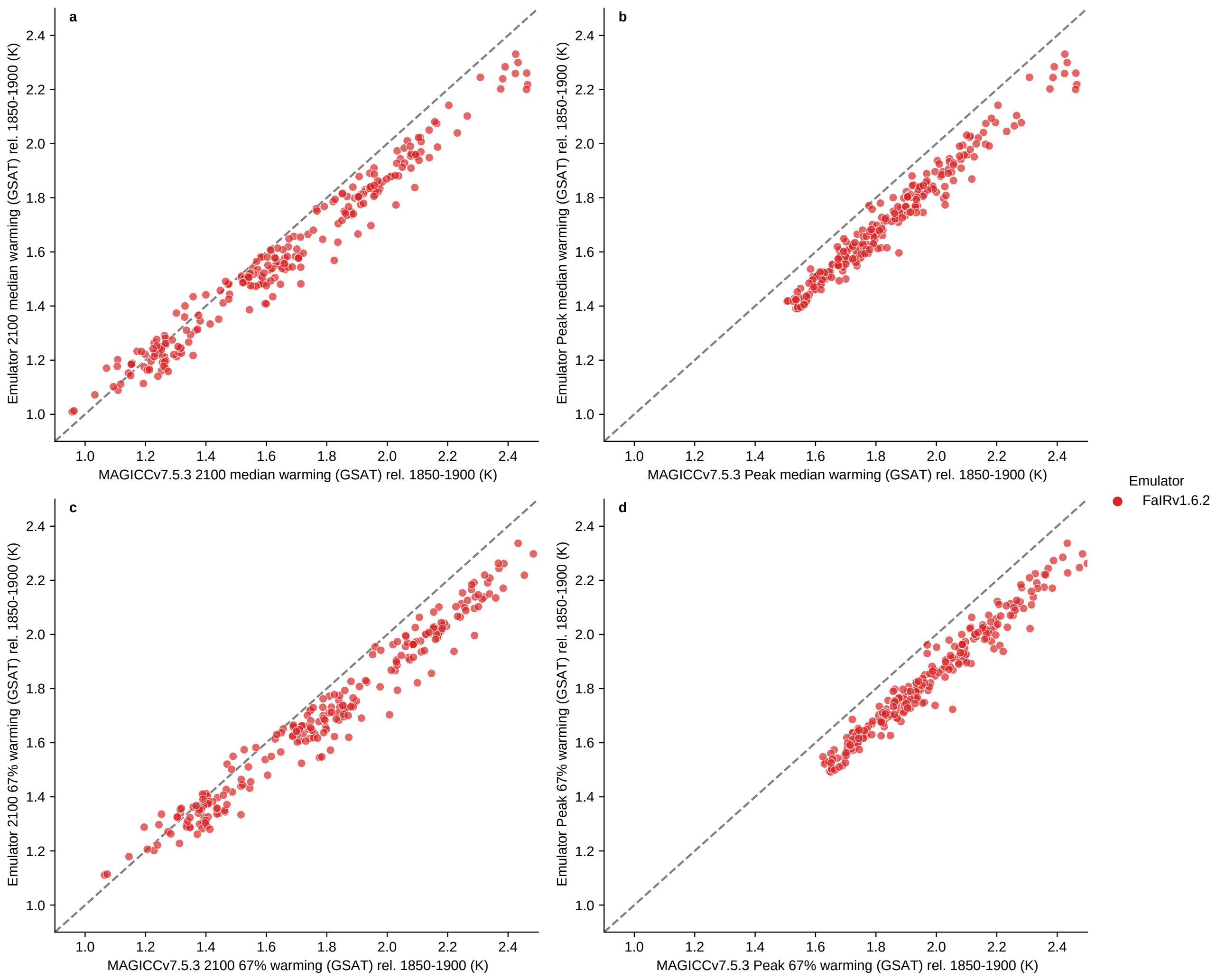

Figure S11. As in Supplementary Figure S5 but for MAGICCv7.5.3 (x-axes) and FaIRv1.6.2.

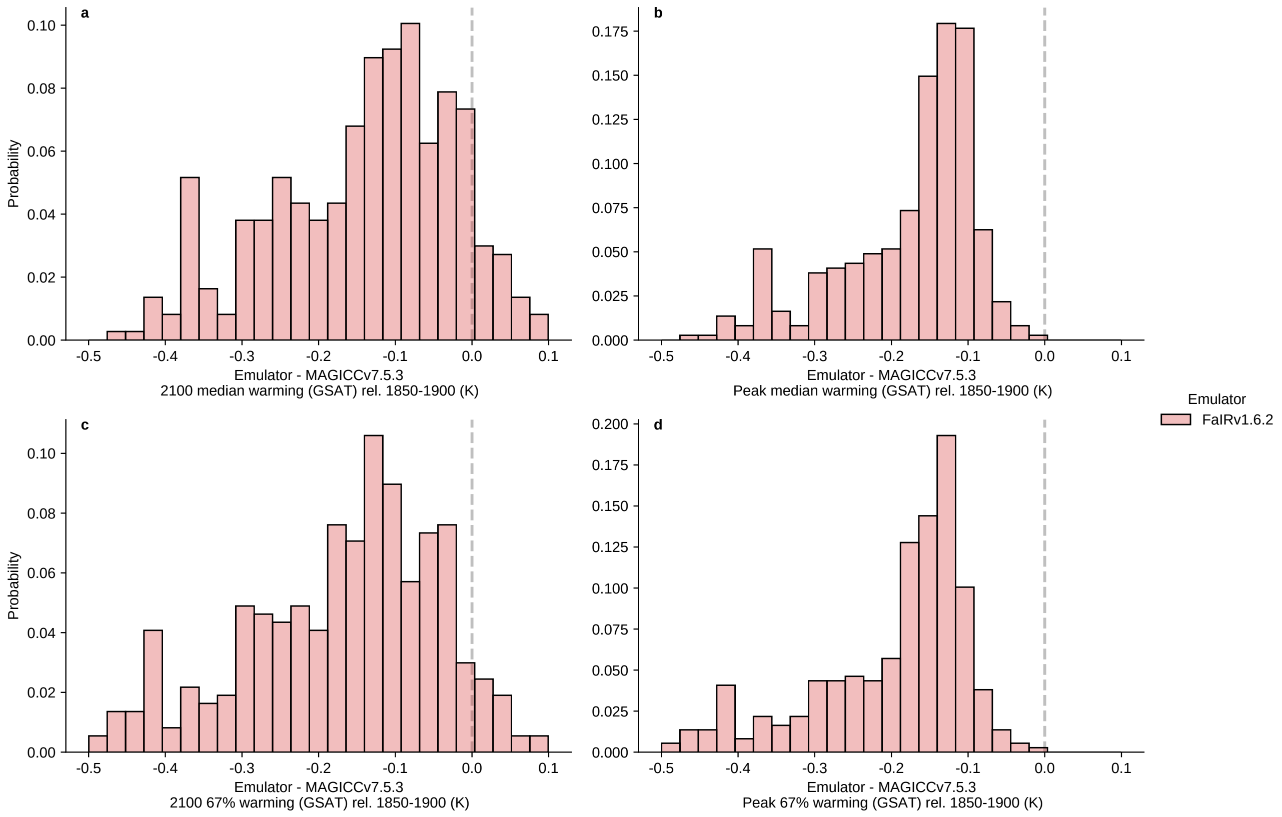

Figure S12. As in Supplementary Figure S6 but for MAGICCv7.5.3 (x-axes) and FaIRv1.6.2. The data is the same as Supplementary Figure S11 but has been plotted as a histogram rather than a scatter plot.

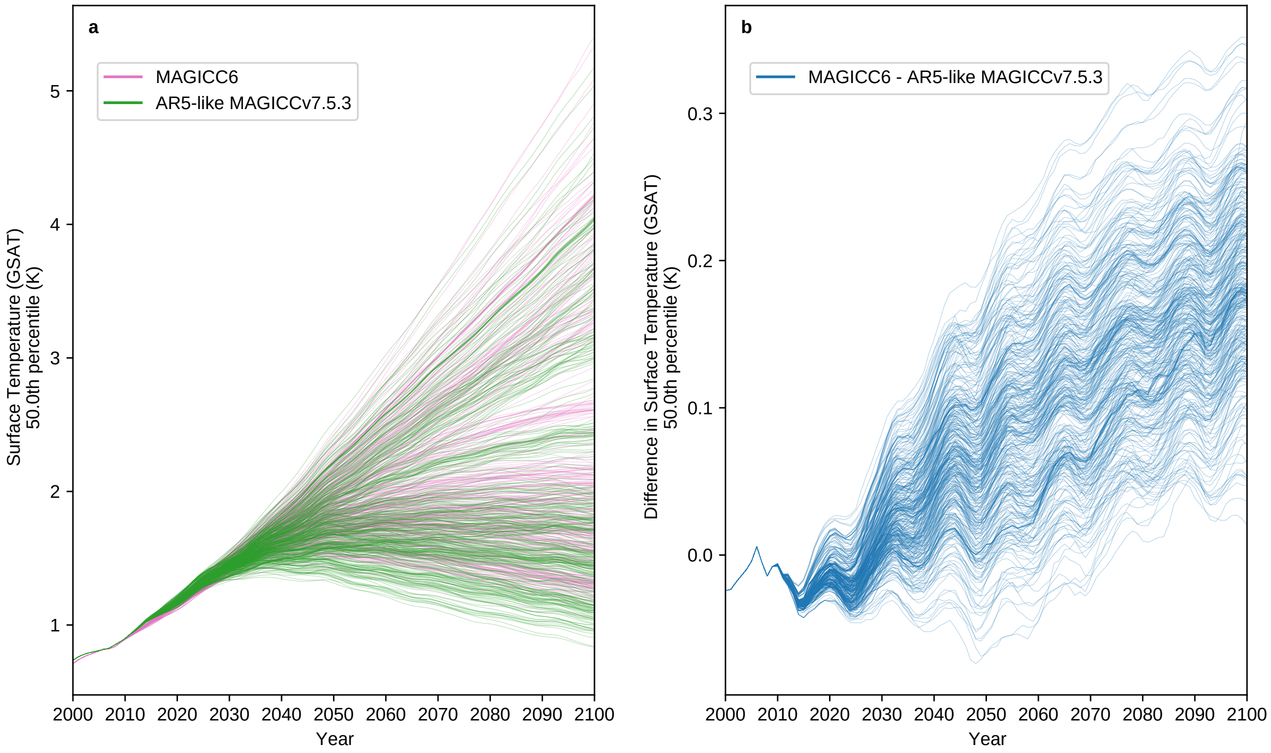

Figure S13. As in Supplementary Figure S7, except for median surface air temperature (GSAT).

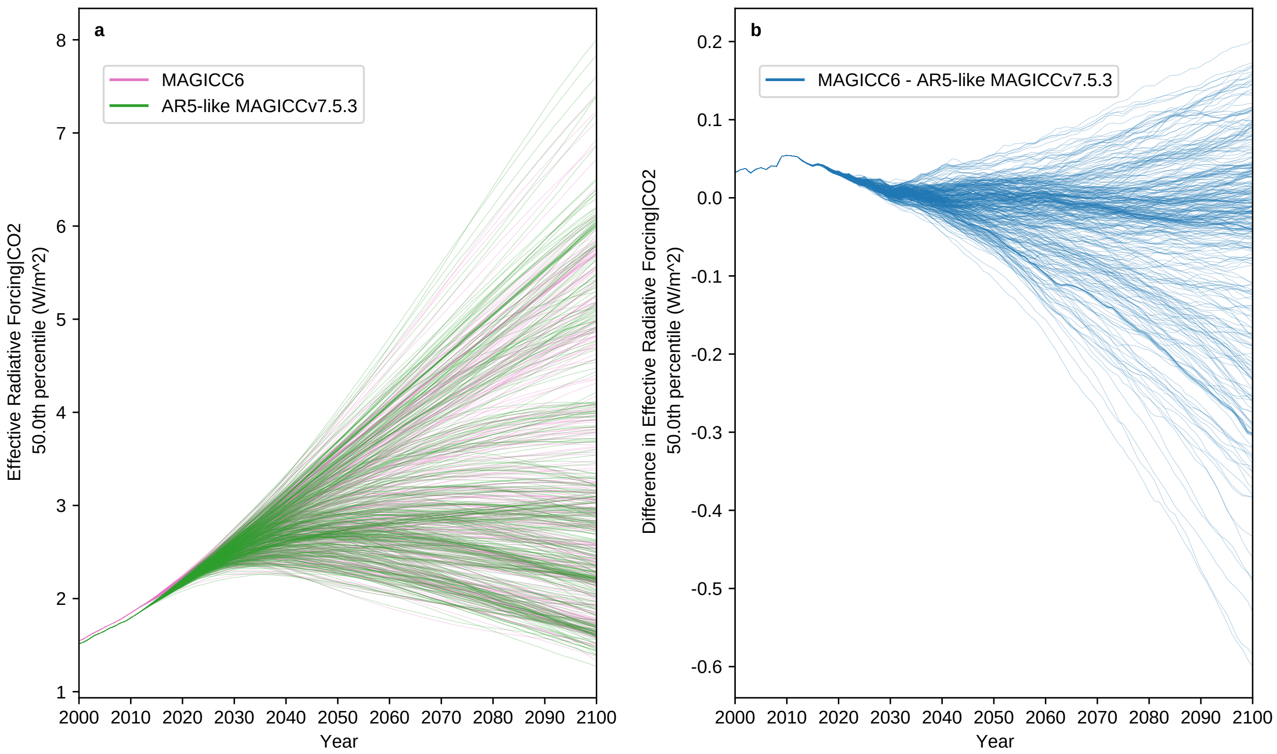

Figure S14. As in Supplementary Figure S7, except for median CO_2 effective radiative forcing.

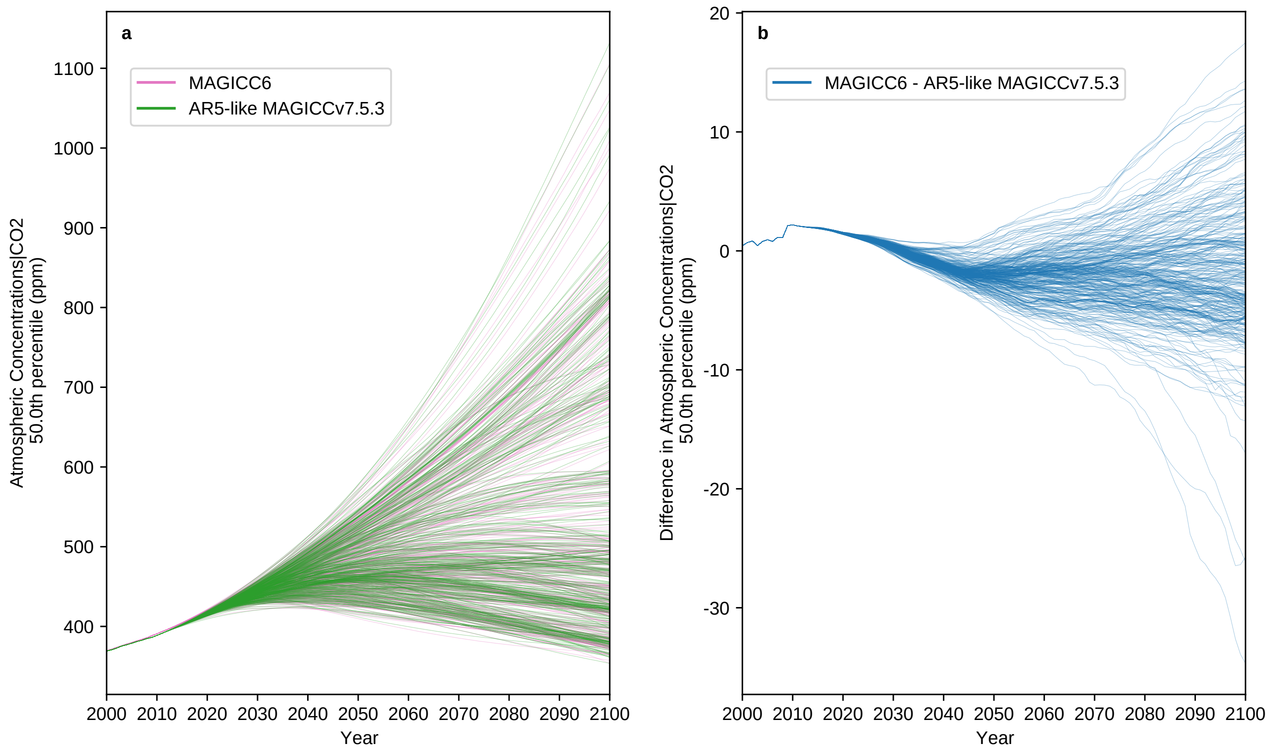

Figure S15. As in Supplementary Figure S7, except for median CO_2 atmospheric concentrations.

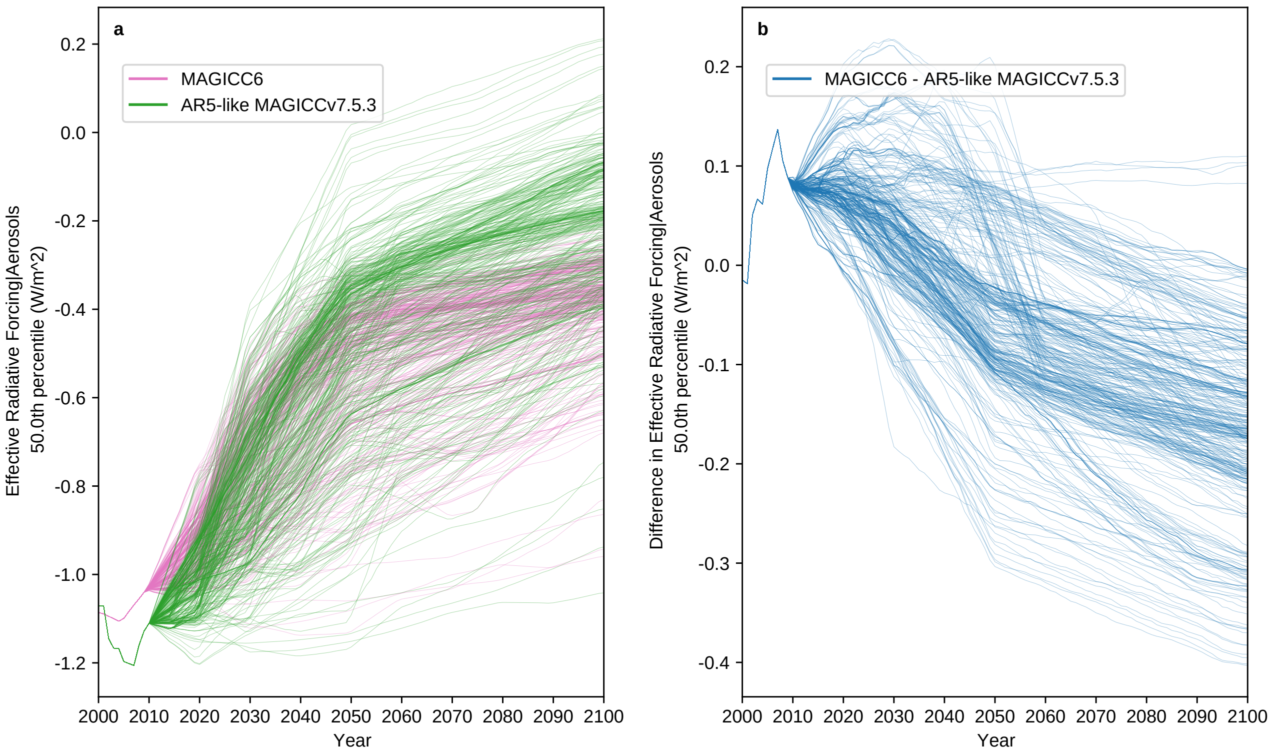

Figure S16. As in Supplementary Figure S7, except for median aerosol effective radiative forcing.

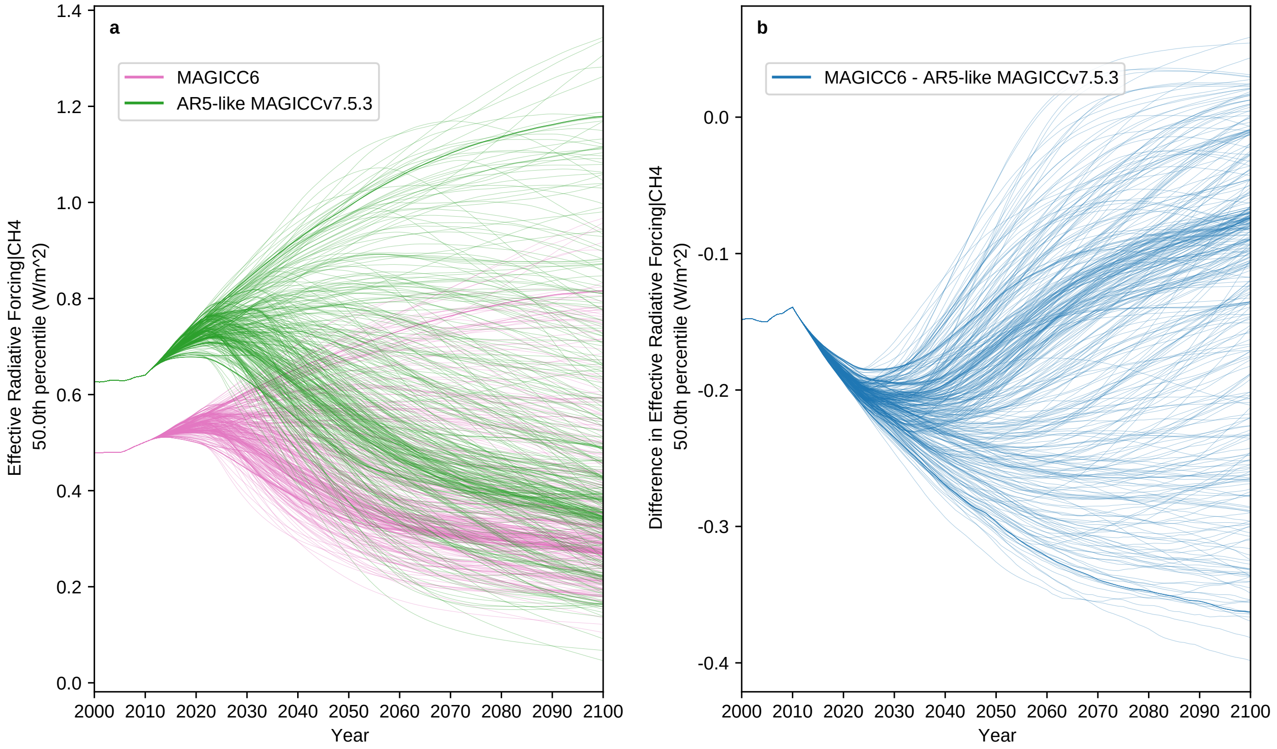

Figure S17. As in Supplementary Figure S7, except for median methane effective radiative forcing.

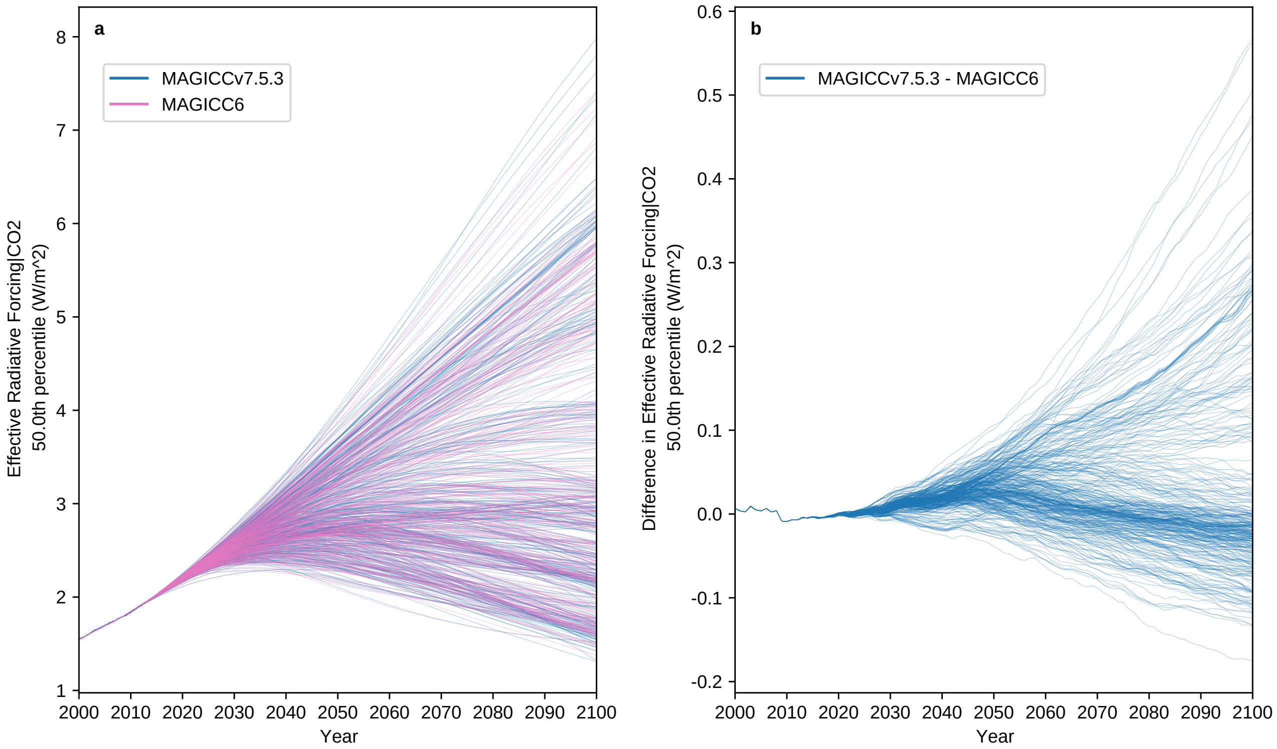

Figure S18. As in Supplementary Figure S9, except for median CO_2 effective radiative forcing.

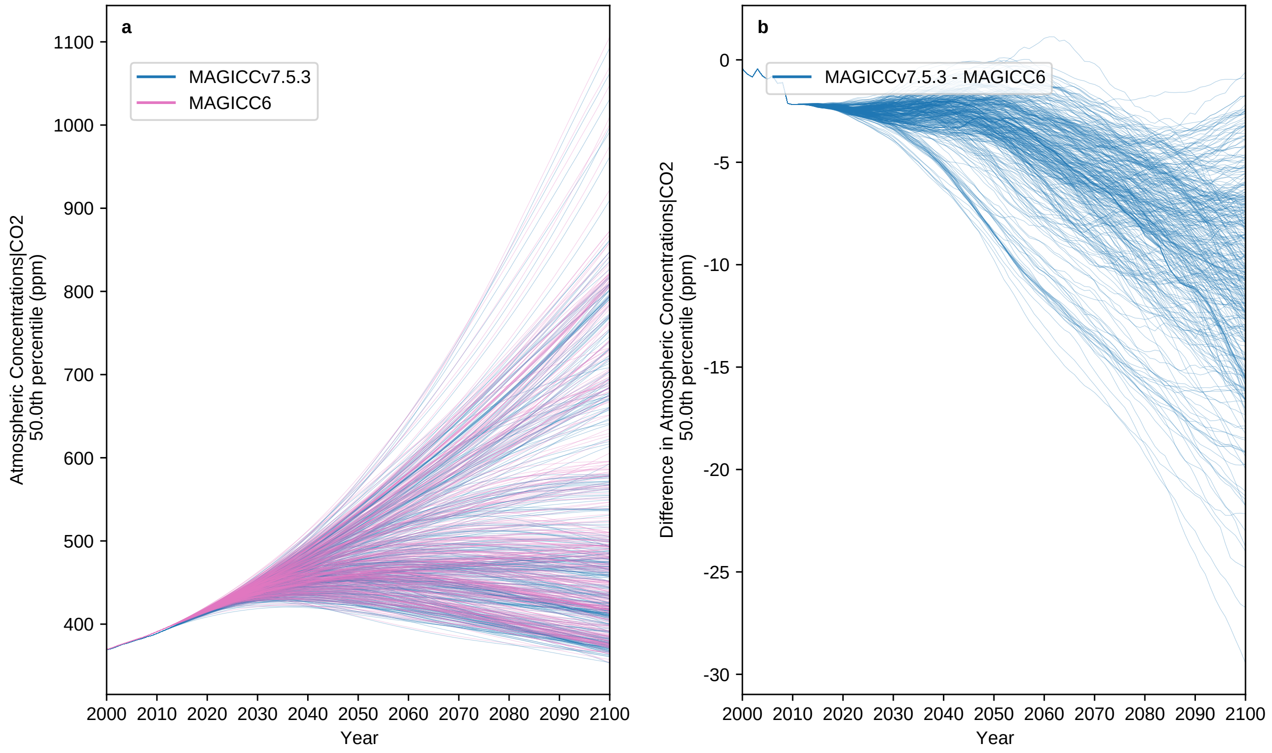

Figure S19. As in Supplementary Figure S9, except for median CO_2 atmospheric concentrations.

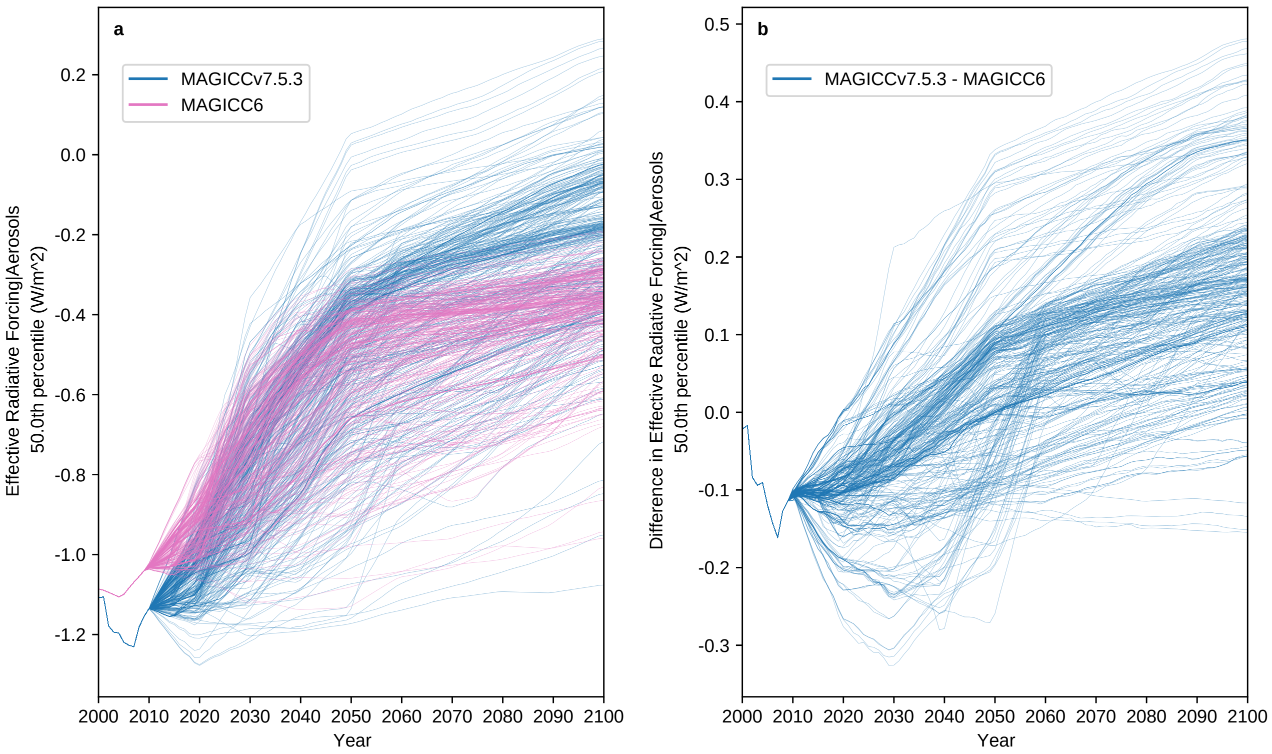

Figure S20. As in Supplementary Figure S9, except for median aerosol effective radiative forcing.

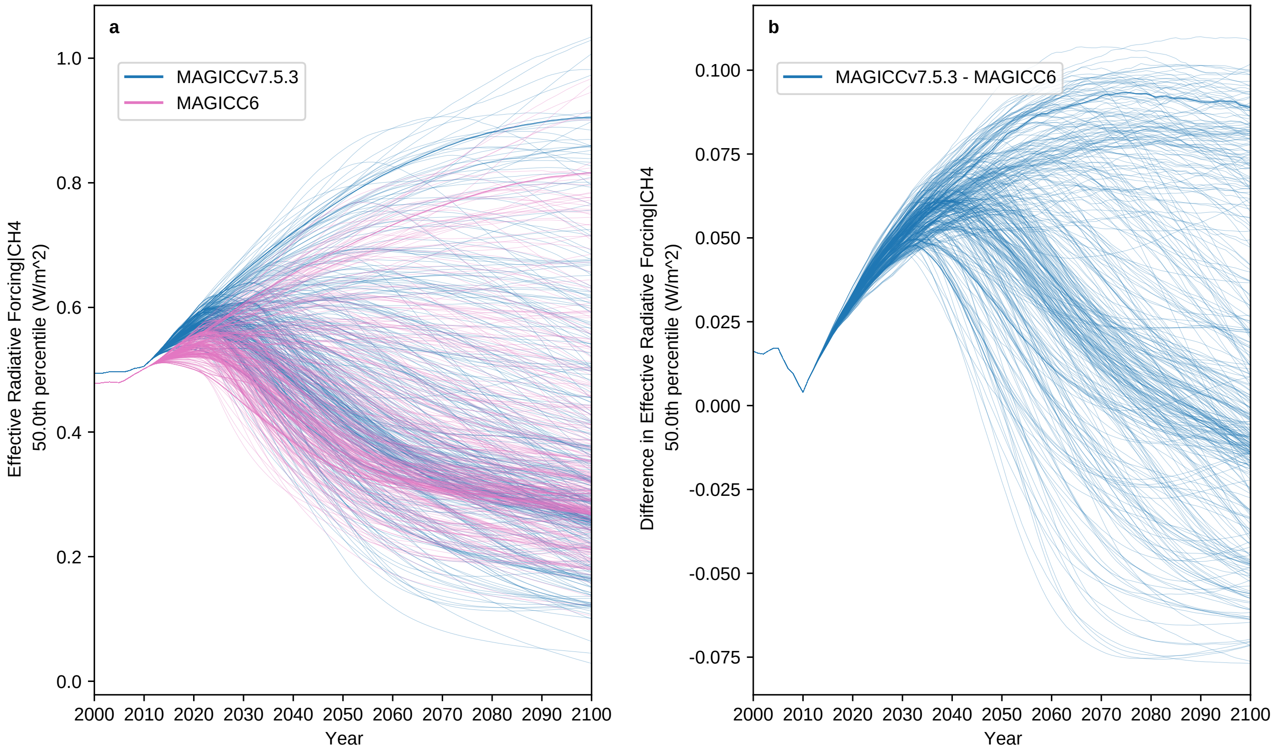

Figure S21. As in Supplementary Figure S9, except for median methane effective radiative forcing.
